# Visibility Restoration: A Systematic Review and Meta-Analysis

**DOI:** 10.3390/s21082625

**Published:** 2021-04-08

**Authors:** Dat Ngo, Seungmin Lee, Tri Minh Ngo, Gi-Dong Lee, Bongsoon Kang

**Affiliations:** 1 Department of Electronics Engineering, Dong-A University, Busan 49315, Korea; datngo@donga.ac.kr (D.N.); 1672885@donga.ac.kr (S.L.); gdlee@dau.ac.kr (G.-D.L.); 2 Faculty of Electronics and Telecommunication Engineering, The University of Danang—University of Science and Technology, Danang 550000, Vietnam; nmtri@dut.udn.vn

**Keywords:** systematic review, meta-analysis, visibility enhancement, haze removal, image dehazing, image defogging

## Abstract

Image acquisition is a complex process that is affected by a wide variety of internal and environmental factors. Hence, visibility restoration is crucial for many high-level applications in photography and computer vision. This paper provides a systematic review and meta-analysis of visibility restoration algorithms with a focus on those that are pertinent to poor weather conditions. This paper starts with an introduction to optical image formation and then provides a comprehensive description of existing algorithms as well as a comparative evaluation. Subsequently, there is a thorough discussion on current difficulties that are worthy of a scientific effort. Moreover, this paper proposes a general framework for visibility restoration in hazy weather conditions while using haze-relevant features and maximum likelihood estimates. Finally, a discussion on the findings and future developments concludes this paper.

## 1. Introduction

**Figure 1:**
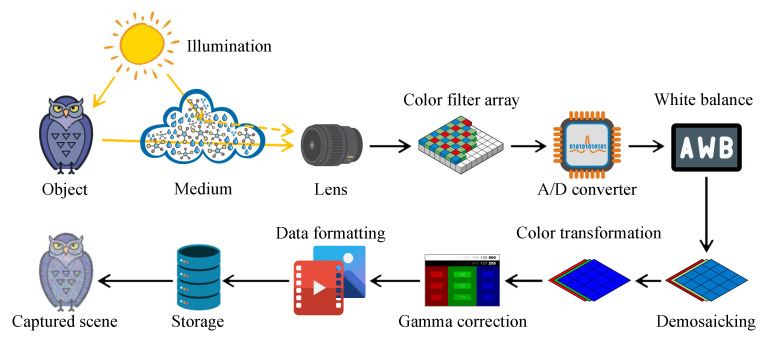
Typical example of digital camera workflow.

Currently, the ubiquity of camera-based systems has increased the demand for high image quality from the end-users. Consequently, the digital camera workflow has become more intricate. Figure 1 exemplifies the in-camera processing flow from the formation to the viewer, following the description of Parulski and Spaulding [1]. Notably, the constituent components and their corresponding order may differ, conforming to camera manufacturers. This section primarily aims to portray the digital camera workflow because Section 2.2 will describe the optical image formation. The end-users hardly perceive the raw image data that are captured by image sensors. Accordingly, a set of algorithms comprising analog-to-digital (A/D) conversion, white balance, demosaicking, color transformation, gamma correction, and data formatting has been instituted. Thus, the image data become perceptible. However, any artifacts or imperfections that are introduced by the foregoing tasks can accumulate and significantly affect the subsequent computer vision applications, such as object recognition, driver assistance, and surveillance.

Hence, visibility restoration is an active research area for alleviating any untoward effects that originate from the image formation and processing pipeline. Earlier, image visibility could be restored by denoising and then reversing the environmental effects. The noise was typically assumed to follow a Gaussian distribution. Meanwhile, the image formation was modeled by adopting theoretical optics. Oakley and Satherley [2] developed a physical model for the contrast enhancement of grayscale images. Subsequently, Tan and Oakley [3] adopted wavelength dependency to extend the previous model to color images. Currently, researchers usually refer to this model as the simplified Koschmieder model. Additionally, with the significant technological advances in image sensors, the noisy effect has substantially diminished, insofar as researchers usually disregard its involvement. Hence, visibility restoration is, highly pertinent to image formation. Despite the fact that there are diverse imaging environments, covering all of them is extremely laborious. Therefore, this study primarily focuses on visibility restoration algorithms for the atmospheric environment.

Recently, the ill-posed nature of visibility restoration has been attracting interest from academia and industry. This is attributable to the potential benefits in consumer photography and computer vision applications. The utilization of visibility restoration algorithms as a pre-processing step in high-level vision tasks (for example, object recognition/localization) is a prime example. Liu et al. [4] demonstrated that the reduction in detection rate was proportional to the haze density, positing that image dehazing is a practical solution for facilitating object recognition algorithms. However, in the development of visibility restoration algorithms, researchers have faced an ever-present under-constrained problem, which is, the number of freedoms exceeds the number of observations. Accordingly, despite the unceasing efforts to circumvent this intractability, visibility restoration remains a challenging problem.

To date, although the myriad algorithms have been put forth to address the aforementioned issue, there are not many studies on systematic reviews of visibility restoration. Indeed, those studies have only covered a few aspects of this many-sided subject. Consequently, it is difficult for researchers to appraise the maturity and ascertain the research trends and future dimensions of the field. It is also necessary to investigate a research agenda for visibility restoration to meet the considerable demand for a generalized and sophisticated algorithm. Hence, a systematic review that collates, classifies, and appraises all of the relevant research results will enable knowledge transfer in the scientific community. As mentioned previously, although there have been a few investigations on visibility restoration, none have included all essential aspects. Liu et al. [4] and Pei et al. [5] investigated the effects of image degradation on object recognition. The results demonstrated that the accuracy declined as haze increased. Applying haze removal algorithms could alleviate this problem to a certain extent, but not much. Li et al. [6] conducted a thorough evaluation, focusing on traditional and deep learning-based dehazing methods. The results demonstrated that the former methods favored human perception, whereas the latter methods favored numerical metrics. Recently, Yang et al. [7] launched a challenge to evoke discussions and explorations regarding exploiting low-level image processing techniques in high-level vision tasks. The results were similar to those displayed by Pei et al. [5], signifying large room for development.

This study conducted a systematic review and meta-analysis according to the PRISMA statement [8] with the primary objective of appraising an extensive corpus of visibility restoration studies and proposing a simple framework for haze removal. As a result of an extensive appraisal, this study classified existing algorithms into three major groups and summarized the relevant advantages and disadvantages. In particular, the results of this systematic review are beneficial for the following individuals in image processing, notably visibility restoration.

- Researchers who require a systematically organized body of knowledge on relevant studies.
- Practitioners who are interested in general knowledge on existing methods and techniques.
- Laypeople who need a readable and understandable review of relevant research.

The remainder of this paper is organized, as follows. Section 2 introduces the PRISMA statement, provides preliminaries on optical image formation, and appraises relevant studies. Section 3 investigates the research agenda, particularly those that are high on the top. Section 4 presents a general framework for haze removal using handcrafted haze-relevant features and maximum likelihood estimates. Finally, Section 5 provides the conclusion.

## 2. Preliminaries

This section first introduces PRISMA—a basis for reporting systematic reviews—and then presents the fundamentals of optical image formation, followed by a general classification of visibility restoration algorithms. The optical image formation lays the foundation stone for virtually all existing methods thus far, and the classification aims to provide practitioners and laypeople with a concise and understandable body of knowledge.

### 2.1. PRISMA

**Figure 2:**
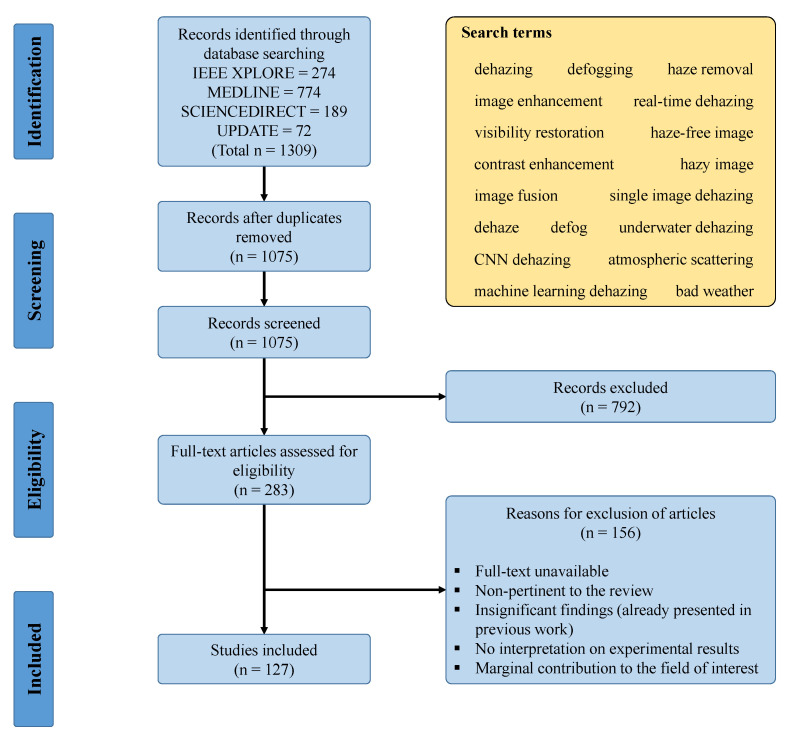
PRISMA flow diagram for the systematic review in this study.

PRISMA is the abbreviation of Preferred Reporting Items for Systematic Reviews and Meta-Analyses, and it is comprised of a checklist and a flow diagram [8]. These two components aid researchers with reporting in systematic reviews and meta-analyses. Figure 2 depicts the four-phase flow diagram with the number of included/excluded studies in each phase.

To begin with, this study obtained 1309 research records through database searching, in which 274 records from IEEE Xplore, 774 records from MEDLINE, 189 records from ScienceDirect, and 72 records that were subsequently added after searching with new keywords. Database searching was conducted using PubMed—a free search engine with Google-like search formulations, and all of the search terms were included in Figure 2. The total of 1309 records then underwent the screening phase, where 234 duplicates were removed, and the remaining 1075 records were uploaded to abstrackr [9] for abstract screening. In this study, the same criteria were used to exclude records in both abstract screening and full-text analysis. Figure 2 also illustrated those criteria. Finally, only 127 studies remained and they were cited in this paper.

### 2.2. Optical Image Formation

There is a universal postulate among the optics community that the image irradiance on a sensor element is a sum of two components: the irradiance that is reflected from the object surface and the irradiance scattered directly to the sensor by the atmospheric aerosol. Notably, this postulate significantly simplifies the actual imaging process. The light that is reflected from the object surface encounters atmospheric aerosols in the path towards the sensor element. Hence, this type of irradiance is also subject to scattering. However, another postulate is that the scattering of reflected light is insignificant in hazy or foggy weather conditions. Therefore, Tan and Oakley [3] defined the irradiance $E_{t}$ at a particular sensor element *k*, as follows:

(1) $$E_{t}\left(k,\lambda ,x\right)=\Omega_{k}S_{0}\left(\lambda \right)R_{k}\left(\lambda ,x\right)exp\left[-\beta_{sc}\left(\lambda \right)d_{k}\left(x\right)\right],$$

where λ denotes the wavelength, *x* denotes the spatial coordinates of image pixels, $\Omega_{k}$ denotes the imaging angle, $S_{0}$ denotes the sky’s mean radiance, $R_{k}$ denotes the reflectance factor, $\beta_{sc}$ denotes the atmospheric scattering coefficient, and $d_{k}$ denotes the distance from the sensor element to the object. Variables with the subscript *k* are pertinent to the sensor element *k*.

The irradiance associated with the light scattered directly to the sensor element (denoted as $E_{b}$) can also be expressed in terms of the scattering coefficient, according to Tan and Oakley [3]. In this context, it is the fraction of light reaching the sensor element after particle-particle collisions between light photons and atmospheric aerosols, as given by

(2) $$E_{b}\left(k,\lambda ,x\right)=\Omega_{k}S_{0}\left(\lambda \right)\left\{1-exp\left[-\beta_{sc}\left(\lambda \right)d_{k}\left(x\right)\right]\right\}.$$

Consequently, the sum of $E_{t}$ and $E_{b}$ denotes the total irradiance $E_{s}$ of the sensor element *k* for a specific wavelength λ, as shown in Equation (3). Notably, the captured scene irradiance is the integration of $E_{s}$ with respect to λ over all imaging wavelengths. It is generally convenient to consider three standard wavelengths that correpond to $\lambda_{red}=650$ nm, $\lambda_{green}=550$ nm, and $\lambda_{blue}=450$ nm, resulting in a red-green-blue (RGB) image. When necessary, another wavelength (for example, near-infrared) can be considered to improve the captured scene irradiance.

(3) $$E_{s}\left(k,\lambda ,x\right)=\Omega_{k}S_{0}\left(\lambda \right)\left\{1+\left[R_{k}\left(\lambda ,x\right)-1\right]exp\left[-\beta_{sc}\left(\lambda \right)d_{k}\left(x\right)\right]\right\}.$$

Recently, researchers have widely exploited the simplified Koschmieder model, in which $E_{t}$ and $E_{b}$ are denoted as direct attenuation and airlight, respectively. In Equation (4), I(k,λ,x) denotes the captured scene irradiance, $J\left(k,\lambda ,x\right)=\Omega_{k}S_{0}\left(\lambda \right)R_{k}\left(\lambda ,x\right)$ denotes the original scene irradiance, $t\left(k,\lambda ,x\right)=exp[-\beta_{sc}\left(\lambda \right)d_{k}\left(x\right)]$ denotes the transmission map (or transmittance), and $A\left(k,\lambda \right)=\Omega_{k}S_{0}\left(\lambda \right)$ denotes the atmospheric light.

(4) I(k,λ,x)=J(k,λ,x)t(k,λ,x)+A(k,λ)[1−t(k,λ,x)].

**Figure 3:**
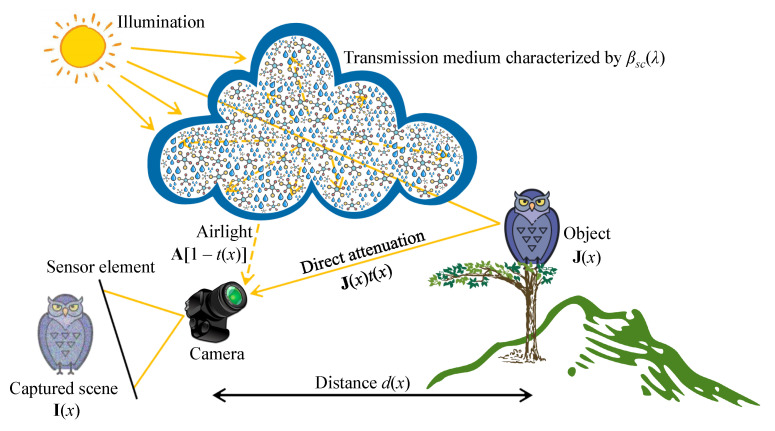
Visual illustration of the optical image formation in the atmosphere.

Moreover, although the digital camera workflow was complex, Grossberg and Nayar [10] discovered that the mapping from scene irradiance to image intensity is uniform across the spatial dimensions of the image. Therefore, Equation (4) can be directly used to represent the digital image formation. Additionally, contemporary researchers often postulate that the dependency of transmittance on wavelengths is relatively weak and it can be ignored. Accordingly, letting I(x)=I(k,λ,x), J(x)=J(k,λ,x), A=A(k,λ), and t(x)≈t(k,λ,x) simplifies Equation (4) to the following:

(5) I(x)=J(x)t(x)+A[1−t(x)],

where the boldface representations are adopted to signify the wavelength dependency. In Equation (5), I is the only observation, whereas J, A, and *t* are the unknowns. Consequently, recovering the original visibility J requires the estimates of A and *t*, causing the ill-posed nature of visibility restoration. Several prior information have been proposed to address this challenging problem, and a wide variety of methods and techniques have been exploited accordingly. Despite such diverse efforts, a few problems, such as color distortion and domain shift, persist, which creates a large room for improvement. Figure 3 concludes this subsection by illustrating the aforementioned optical image formation in the atmosphere, in which each constituent component has been labeled.

### 2.3. General Classification

**Figure 4:**
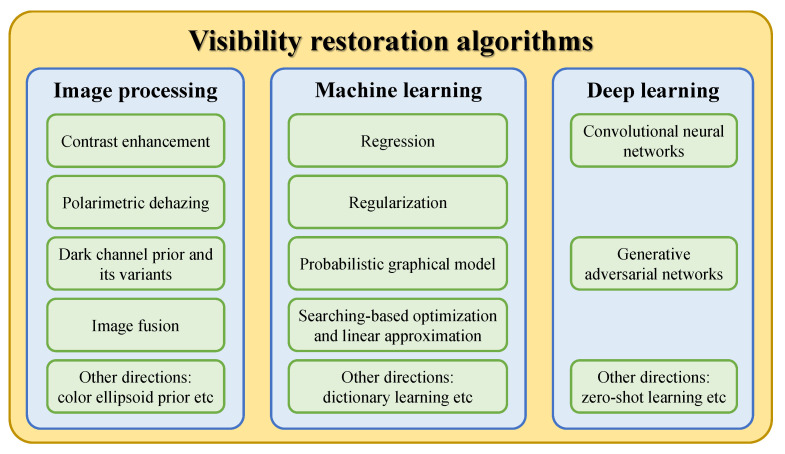
General classification of visibility restoration algorithms.

This subsection collates studies on the visibility restoration field and broadly classifies them into three main categories: image processing, machine learning, and deep learning techniques, as illustrated in Figure 4. In most existing studies, researchers often categorize visibility restoration algorithms according to the number of input images into single-image and multiple-image algorithms. Hence, this study approaches the categorization from a different perspective to arrive at the aforementioned three categories, aiming to bring a new dimension to the field of interest. More specifically, this study appraises visibility restoration algorithms by considering the practicality of deploying them in real-world applications. In this context, the first category—image processing—consists of hand-engineered methods that are framed using domain knowledge about image degradation. Exemplars of such methods are contrast enhancement, image fusion, and morphological operations. The second category—machine learning—typically involves exploratory data analysis to obtain statistical regularities of relevant datasets. Prime examples of this are maximum likelihood estimates, support-vector machines, and *k*-nearest neighbors algorithms. The last category—deep learning—refers to the increasing exploitation of deep neural networks in image processing tasks and it is exemplified by convolutional neural networks and generative adversarial networks. Although each category is not clearly distinguishable from one another, the classification in this study is deemed germane to the recent applications of constituent methods. Coming subsections will describe those three categories in more detail.

#### 2.3.1. Image Processing

**Contrast enhancement and polarimetric dehazing:** initially, contrast enhancement was a viable solution to visibility restoration because the image contrast considerably influenced the human perception of image quality. Kim et al. [11] proposed a block-overlapped histogram equalization method operating on image sequences that significantly enhanced video visibility on mobile phones and security cameras. Oakley and Satherley [2] devised a physical model describing contrast degradation in a turbid atmosphere. They also proposed a compensation scheme using a temporal filter to address the exponential reduction of the signal-to-noise ratio when processing the image sequences. Although these early attempts demonstrated promising results in the near field, they shared a common problem pertaining to noise amplification in the far field. Recently, Kim et al. [12] adopted the contrast stretch concept to estimate the scene radiance’s saturation that was directly used to derive the medium transmittance. Moreover, they exploited the white balance technique to remove the color veil and, thus, laid the extended applicability to yellow-dust-degraded images. This method showed great promise in real-world applications, owing to its low complexity, good dehazing performance, and versatility.

Schechner et al. [13] developed a polarimetric dehazing model from a postulate about the sole polarization of the airlight. This model required at least two images that were captured under different degrees of polarization, which were used to reduce the number of freedoms in the optical hazy image formation. In general, image dehazing based on the polarimetric model leveraged two images, including p-polarized and s-polarized images, which were pertinent to the incident light parallel and perpendicular to the incidence plane, respectively. Fade et al. [14] instituted an experimental implementation for polarimetric imaging that resulted in the previous two images. In order to improve the pioneering work of Schechner et al. [13], Fang et al. [15] amended the existing postulate by considering the polarization of the object and presented a decorrelation-based scheme for estimating the airlight. Conversely, Liang et al. [16] and Liu et al. [17] retained the postulate of Schechner et al. [13] and focused on addressing its limitations. Liang et al. [16] adopted the distribution analysis of the angle of polarization to obtain an accurate estimate of the airlight, whereas Liu et al. [17] adopted image decomposition to dehaze the base layer and emphasize the detail layer. Although these methods surmounted the far-field noise amplification problem, they were not widely deployed, owing to the burdensome configuration of the experimental equipment. Zhang et al. [18] presented a field-programmable gate array (FPGA) prototype for facilitating the application of polarimetric dehazing; however, this study did not report hardware synthesis results, causing difficulties in appraising its practicality. Furthermore, those involving airlight estimation also lacked generality, because they required the presence of sky areas to function correctly. Recently, Qu and Zou [19] and Liang et al. [20] attempted to overcome this problem, but the results were unimpressive.

**Figure 5:**
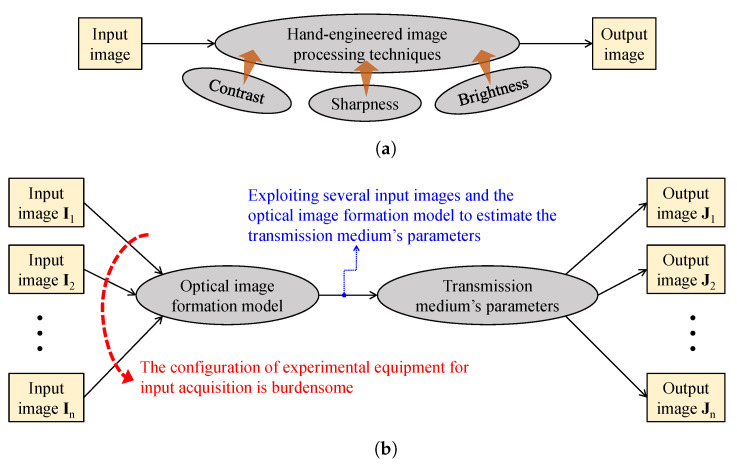
Simplified block diagrams of: (**a**) contrast enhancement and (**b**) polarimetric dehazing approaches in visibility restoration.

Figure 5 provides simplified block diagrams of the aforementioned two approaches. In Figure 5a, visibility restoration cthroughontrast enhancement is typically hand-engineered by investigating image contrast, sharpness, and brightness, because weather-related image degradation has manifest effects on those image features. Therefore, this approach often results in a noticeable improvement in image quality; however, the degradation persists, owing to the ignorance of degradation sources. In Figure 5b, the polarimetric dehazing approach addresses the ill-posed nature of visibility restoration by utilizing several input images that were taken under different polarization degrees. With sufficient images and postulates regarding the airlight, acceptable estimates of parameters that characterize the transmission medium are attainable, enabling the restoration of clean images. Nonetheless, this approach has one major disadvantage—the burdensome configuration of experimental equipment for input acquisition.

**Dark channel prior and its variants:** the discovery of the dark channel prior (DCP) through an extensive observation of haze-free outdoor images by He et al. [21] proved to be a significant turning point. This prior note was widely publicized and used in diverse applications in computer vision. For example, Chiang and Cheng [22] utilized the DCP to estimate the depth map, which was used for background/foreground segmentation to detect and remove artificial light sources in underwater image enhancement. In addition, Gu et al. [23], Wang et al. [24], and Ruiz-Fernandez et al. [25] successfully exploited the DCP in clinical applications, such as laparoscopic surgery and digital radiography. The DCP states that the image local patches possess extremely dark pixels whose intensity is approximately zero in at least one color channel. The rationale behind this prior is the colorfulness of outdoor objects, except for the sky region, whose color intensities are high in all channels. Hence, although the DCP generally provides accurate transmittance estimates, it may fail and, consequently, produce artifacts in sky regions. Additionally, because the DCP was a patch-based prior, He et al. [21] adopted soft matting [26] to refine the estimated transmittance, inducing the high complexity limitation. Accordingly, the DCP has aroused keen interest among contemporary researchers, resulting in its improvements in several dimensions.

He et al. [27] proposed a guided image filter (GIF), which is an excellent edge-preserving smoothing filter, to replace the computationally expensive soft matting. GIF considerably shortened the processing time at the cost of a certain degree of degradation. Subsequently, Li et al. [28] developed a weighted GIF (WGIF) by introducing an edge-aware weighting scheme into the existing GIF. They also devised a DCP-like dehazing approach, where WGIF was used instead of GIF to refine the estimated transmittance. Li and Zheng [29] later improved WGIF by a globally guided image filter (G-GIF), which embodied global structure transferring and global edge-preserving smoothing techniques. A DCP-like dehazing method that they developed was equipped with sky-awareness and fine detail preservation. Sun et al. [30] furthered the work of Li et al. [28] by exploiting the salient features of the guidance image. They adopted the steering kernel, whose coefficients were determined by singular value decomposition and local gradient matrix, to learn the edge direction from the guidance image in an adaptive manner. Thus, WGIF with steering kernel demonstrated a better dehazing performance than GIF and WGIF, at the cost of extended processing time. However, the presented results in the foregoing methods appeared to be limited for a comprehensive appraisal.

Moreover, Yeh et al. [31] estimated a pixel-wise dark channel by eliminating the patch-based minimum operation. Subsequently, they exploited the bilateral filter to refine the estimated transmittance, slightly decreasing the algorithmic complexity. Nonetheless, the results appeared to be slightly over-saturated. Yeh et al. [31] also proposed the extreme channels, which was, dark and bright channels, for atmospheric light estimation. Sun et al. [32] later exploited this idea to estimate the airlight utilizing morphological operations and bilateral filtering. Despite the significantly restored visibility, the color shift persisted in the sky region. Morphological operations were also leveraged by Salazar-Colores et al. [33] to replace the soft matting in the original DCP, substantially reducing the algorithmic complexity and memory usage. Notably, Li and Zheng [34] extended DCP in a creative direction. They postulated that the variation within the dark channel was small; and, this postulate held true for the sky region. The WGIF was subsequently employed to decompose the dark channel into base and detail layers. The transmittance was estimated from the base layer, and an adaptive compensation scheme was devised, lest the far-field noise amplification occurred. However, this method is computationally expensive and it may result in an inaccurate estimate of the transmittance.

Other methods focused on improving the DCP, insofar as the refinement step could be eliminated. Tarel and Hautiere [35] devised a novel method for a faster estimation of the airlight using an edge-preserving smoothing technique, called the median of the median along a line. Although this method significantly shortened the processing time, it introduced halo artifacts in fine details in the image. Kim et al. [36] subsequently addressed this drawback by improving the edge-preserving smoothing technique through the modified hybrid median filter. However, this method left background noises unfiltered in the smooth region, which might be perceptually unfavorable. Gibson et al. [37] replaced the patch-based minimum operation in the DCP with a median filter, substantially declining the computational load. Although halo artifacts ceased to occur after dehazing, the color shift persisted in the sky region. Amer et al. [38] proposed the optimized DCP that could be calculated from the Gaussian-filtered standard-deviation-subtracted version of the input image. Because this method was developed for underwater image enhancement, it was not easy to compare with the DCP. However, the optimized DCP eliminated the refinement step; hence, it offered a conspicuous advantage in terms of processing time.

**Figure 6:**
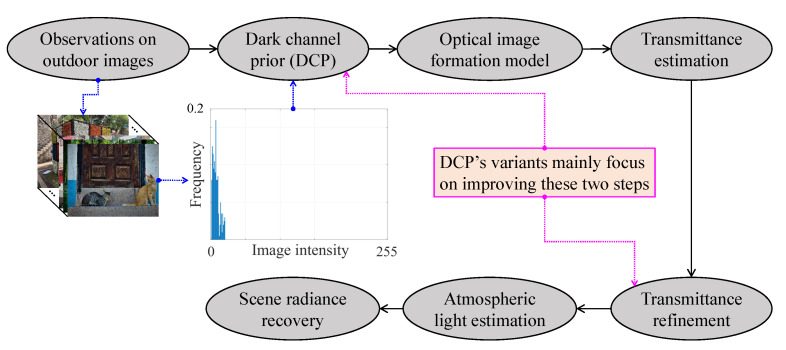
Simplified block diagram of dark channel prior-based visibility restoration methods.

Figure 6 summarizes the essential steps that are involved in DCP-based visibility restoration algorithms. Beginning with the DCP’s derivation from observations on real-world images, the medium transmittance can be estimated while using the postulate that the haze-free image’s dark channel approximates to zero. Transmittance refinement is then for compensating for any untoward effects that are caused by the previous postulate. Finally, the haze-free image can be restored while using the transmittance and the atmospheric light estimates. Subsequently, DCP’s variants improve the DCP’s postulate and the transmittance refinement to reduce post-dehazing artifacts and algorithmic complexity.

**Image fusion:** the aforementioned methods were prone to noise amplification and patch-based artifacts; therefore, they exploited a computationally expensive refinement step or compensation scheme. Accordingly, the researchers approached image dehazing from the image fusion perspective to circumvent the estimation process. Ancuti and Ancuti [39] pioneered the work by utilizing multiscale image fusion to restore the hazy image visibility. White-balanced and contrast-enhanced versions of the hazy image were the inputs for image fusion, where the corresponding weight maps were derived from the image luminance, chromaticity, and saliency. The multiscale fusion was conducted conforming to the Laplacian pyramid representation to avoid post-fusion artifacts. Although the results were impressive, the up- and down-sampling operations in the multiscale fusion did not favor the hardware realization for real-time processing because of a large number of requisite image buffers and line memories. Ngo et al. [40] addressed this problem by demonstrating that the performance gap between single and multiscale fusions was insignificant, while considering the small patch size (for example, 3×3). This finding favored a real-time hardware accelerator for haze removal that is capable of handling high-quality 4K images.

Choi et al. [41] furthered the work of Ancuti and Ancuti [39] by using an additional fog-aware contrast-enhanced image as a third input to the fusion. Moreover, perceptual fog density, luminance, and contrast were used in addition to the original three features to derive the weight maps. Therefore, this increase in the computational cost slightly improved the fused image. Image fusion could also be exploited to obtain the accurate estimates of the transmittance and airlight. Guo et al. [42] estimated two transmission maps that were based on the boundary conditions and then fused them. The final transmittance yielded a strong enhancement for the sky region and weak enhancement for the rest of the image. Ancuti et al. [43] recently extended their work to consider the heterogeneous lighting conditions of nighttime scenes. Their method estimated the airlight with two different patch sizes pertaining to daytime and nighttime scenes, respectively. Subsequently, the corresponding dehazed images, coupled with the discrete Laplacian of the original image, were fused to produce a clean image. These methods demonstrated impressive results, but they were computationally expensive.

Another branch of image fusion-based dehazing fuses the RGB image with a near-infrared (NIR) image. The rationale behind this approach is pertinent to the wavelength-dependency of the hazy image formation. As opposed to visible light waves, the NIR wavelength exhibits less absorption and scattering losses and it retains more structural information. Liang et al. [44] improved polarimetric dehazing by applying this paradigm to an NIR image. The result obtained was fused with that of the RGB image to improve the restored visibility. Similarly, Zhou et al. [45] proposed a multiscale fusion of RGB and NIR images for nighttime vision enhancement, in which the RGB image underwent a pre-enhancement step that was based on high dynamic range (HDR) compression. Despite the impressive performance in terms of restored visibility, these methods faced a practical challenge of the burdensome configuration of the experimental equipment. Jee and Kang [46] presented an exciting idea regarding color reconstruction from an RGB-NIR multispectral filter array. This method could be exploited to pre-enhance the hazy image by leveraging structural information from the NIR image, favoring the design of the subsequent dehazing algorithm.

**Figure 7:**
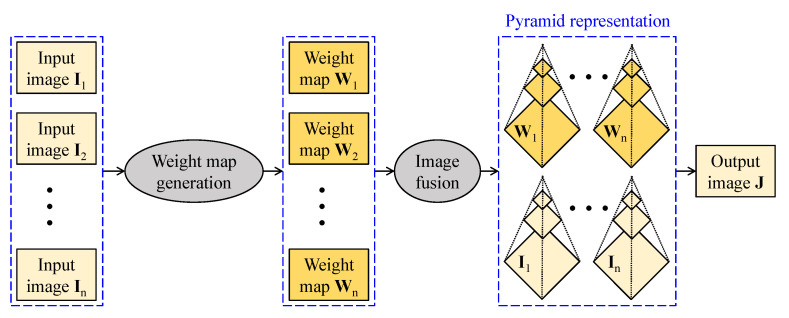
Simplified block diagram of image fusion-based visibility restoration methods.

Figure 7 sketches out the computational flow of image fusion-based visibility restoration algorithms. Input images to the fusion can be either real or artificial. In this context, the real images are typically acquired from a single camera at different polarization degrees or from a set of cameras whose constituents are sensitive to different light spectra. In contrast, artificial images are generated from a single input using diverse image processing techniques. Given input images, corresponding weight maps are then derived according to the fusion purpose, and the fusion is typically conducted at multiple scales using pyramid representation. This approach circumvents the computation-intensive estimation of transmittance and atmospheric light; hence, it offers satisfactory performance while retaining a fast processing rate.

**Other directions:** Deng [47] presented a generalized model for logarithmic image processing, which is known as GLIP, pertaining to gigavision sensors. This imaging device possesses a logarithmic response function, favoring HDR-relevant applications. The GLIP model lays a solid foundation for several low-level image processing tasks, such as contrast enhancement and tone mapping, which benefit image dehazing considerably. Zhang et al. [48] developed a biologically inspired retina model, which comprised three types of cells, for image dehazing. The bipolar cell approximately removed the low-frequency constituents of haze. The amacrine cell enhanced the image contrast to compensate for the loss of details. Finally, the retinal ganglion cell refined the local haze removal and enhanced image details. Luo et al. [49] proposed a hybrid method leveraging the filtering approach of Tarel and Hautiere [35] and image fusion. The use of the bilateral-of-bilateral-grid filter to replace the median filtering technique was a noticeable difference. In addition, luminance fusion was conducted in the gradient domain to compensate for the color infidelity problem that is induced by the dehazing process. The reported results demonstrated a considerable improvement when compared to that of the methods of Tarel and Hautiere [35] and Ancuti and Ancuti [39].

Recently, Bui and Kim [50] developed a statistically robust prior, known as the color ellipsoid prior (CEP). The CEP estimated the transmittance from the ellipsoid geometry that modeled tight clusters of hazy pixels in the RGB space. Because the shape of the ellipsoid was determined by the measurable deviations of clusters, the minority of noisy pixels was effectively averaged and did not affect the estimation accuracy. Additionally, they embedded fuzzy segmentation into the transmittance estimator to suppress the halo artifacts. This method was considerably fast and it exhibited impressive dehazing performance. Furthermore, Mandal and Rajagopalan [51] reduced hazy image formation to a patch-based equation while using multiplicative and additive factors. Subsequently, they assumed that the scene depth changed gradually within an adjacent neighborhood around local patches and exploited this assumption to estimate the two factors. Despite the impressive results and high versatility, the algorithmic complexity was exceptionally high, and ringing artifacts might be observable.

**Figure 8:**
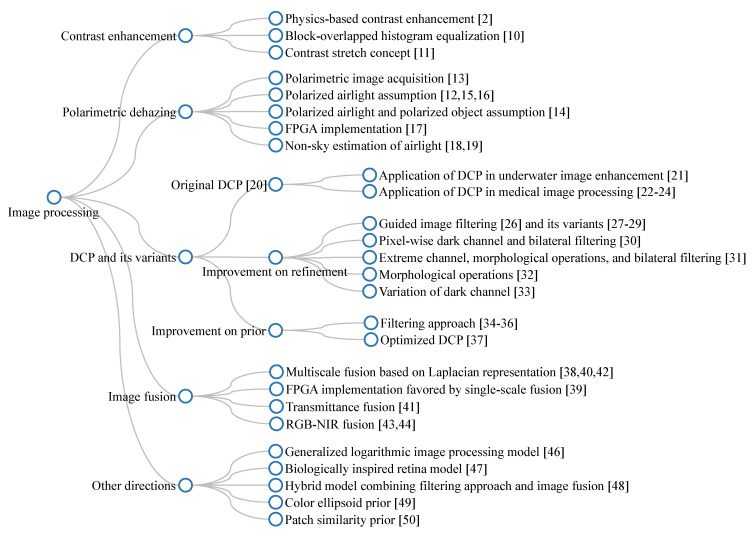
Branching diagram summarizing image-processing-based dehazing algorithms.

Figure 8 depicts a branching diagram that summarizes the aforementioned introduction of dehazing algorithms utilizing image processing techniques. The label of each node has been assigned, such that it is consistent with the occurrence order in the main text. Hence, this diagram is beneficial to laypeople who are interested in an overall overview of existing techniques.

#### 2.3.2. Machine Learning

**Regression analysis:** scientific advances in imaging and memory technologies facilitate the acquisition and storage of a considerable amount of image data. Hence, a detailed observation of the collected data may yield statistically significant regularities, which are exploitable for visibility restoration. Tan and Oakley [3] employed maximum likelihood estimates (MLE) to estimate the atmospheric light and medium transmittance. In this context, they assumed that the terrain reflectance followed a uniform, Gaussian, or surveyed distribution. The last type of distribution was obtained from national surveys, and hence its name. Indeed, this early attempt was prone to noise amplification problems. Zhu et al. [52] also exploited MLE to estimate the coefficients of their proposed model, which calculated the scene depth as a linear combination of the image saturation and brightness. This model was rested on the color attenuation prior (CAP) discovered through extensive observations of hazy images. Although the CAP was a fast and straightforward solution, the results were affected by color distortion, background noise, and post-dehazing false enlargement of white objects. These limitations were addressed by Ngo et al. [53,54] through adaptive weighting, low-pass filtering, and atmospheric light compensation, respectively. Nevertheless, CAP-based dehazing algorithms appeared to be ineffective against dense haze scenes.

Tang et al. [55] exploited random forest regression to estimate the transmittance from a set of multiscale features, including the dark channel, locally maximum contrast, hue disparity, and locally maximum saturation. This method partly alleviated the color distortion in the sky region, and the performance could be considerably improved by considering more haze-relevant features. However, transmittance inference utilizing random forest regression was extremely time-consuming. Jiang et al. [56] modeled the optical depth as a second-order polynomial combination of seven haze-relevant features. Subsequently, they leveraged sensitivity and error analyses to reduce the number of employed features from seven to three, including the dark channel, product of saturation and value, and chroma. Nonetheless, the results tended to be bluish. Meanwhile, Lee et al. [57] reformulated the visibility restoration problem to consider demosaicking artifacts and sensor noises in a joint optimization. Total least squares regression was utilized to solve the optimization and improve the robustness to noises. However, this method was computationally expensive, and the results appeared to be mildly blurred.

Furthermore, Gu et al. [58] proposed a non-reference image quality assessment (IQA) metric and employed it as a quality measure to guide a histogram modification-based dehazing algorithm. Their IQA metric was an output of a regression model, whose inputs were 17 image features pertinent to contrast, brightness, and sharpness, to name but a few. This method was indubitably time-consuming because of the large number of employed features, and the results were over-enhanced. Peng et al. [59] generalized the DCP using the depth-dependent color change assumption, in which a three-bit indicator was employed to signify whether the color intensity increased or decreased as the depth increased. Subsequently, they assumed that the color intensity was linearly correlated with the scene depth and it adopted linear regression to estimate the indicator and the significance weighting factor, which were utilized to estimate the scene depth. Despite the good results and broad applicability, the assumption on the linear relationship was easily broken, resulting in failures for images with heterogeneous lighting conditions. Recently, Raikwar and Tapaswi [60] estimated the medium transmittance using the difference of minimum color channels, which was modeled by the bounding function. Next, they adopted a supervised learning method that was fundamentally similar to MLE to estimate this function. However, the results were affected by color distortion in the sky region.

**Regularization:** simple regression techniques are prone to overfitting, which is, the machine learning model is strictly fit to the training dataset. Thus, it is highly likely to yield a high error rate on future unseen data. Accordingly, regularization, another form of regression, was developed to avoid the risk of overfitting. In this context, a regularization term was added to the loss function to reduce the model variance, thus increasing the ability to capture the true properties of the training dataset, notably those containing noisy data. Schechner and Averbuch [61] adopted adaptive regularization to address the noise amplification problem persisting in their previous work [13]. The employed regularization term was the discrete Laplacian of the scene radiance that was modified by a depth-dependent weighting factor. The qualitative results demonstrated that the far-field noise was suppressed significantly, but not completely. Li et al. [62] exploited relative total variation (TV) regularization, in which the regularization term was the TV measure, to improve the estimation accuracy of the medium transmittance. Because they leveraged the extreme channels to estimate the transmittance, TV regularization was adopted to remove the textual information captured by the minimum and maximum operations, thereby effectively preserving depth information.

Furthermore, Kim et al. [63] utilized a stereo image pair to estimate the transmittance. In this context, they adopted the combined local-global approach with total variation to predict the disparity map, which positively correlated with the transmittance, owing to its inverse relationship with the scene depth. The estimated transmittance was also refined iteratively while using the temporarily dehazed result. Consequently, this method was computationally expensive and inappropriate for real-time processing. Similar to image-fusion-based dehazing algorithms utilizing NIR images, Son and Zhang [64] proposed a near-infrared coloring method that was applicable to haze removal. They adopted regularization to devise a linear mapping model for creating a new NIR image, analogous to its RGB counterpart. Subsequently, color transfer was performed to obtain an RGB image that possessed contrast and details of the captured NIR image. Li et al. [65] developed a robust Retinex model by considering additive noise and formulated a regularized optimization for low-light image enhancement. They utilized the TV measures of the illumination and reflectance as regularization terms and adopted the alternating direction minimization technique to solve the optimization. They also demonstrated that their proposed model could be extended to other visibility restoration tasks, such as image dehazing; however, the results suffered from the loss of details.

Similar to Li et al. [62], Liu et al. [66] adopted non-local TV regularization to preserve the depth information while smoothing textual details in the transmittance refinement step. While using the refined transmittance, they devised an adaptive regularized model for scene radiance recovery. Although the dehazing performance was impressive, it was reliant on the initial estimates of the transmittance. In another approach, Pan et al. [67] utilized the dark channel’s sparsity as a regularization term, and they devised a linear approximation technique to solve the induced non-convex optimization problem. However, the results were slightly bluish, and the processing time was prolonged. Dong et al. [68] estimated the medium transmittance by optimizing the local contrast regarding the information loss (that is, the number of truncated pixels owing to underflow and overflow). They also proposed an exciting idea of leveraging the network of local traffic cameras to reduce the processing time. In this context, one camera was configured as the calibration camera, whose objective was to calculate the initial transmittance. This value was applied to other cameras in the same network, which considerably reduced the processing time. Recently, Wu et al. [69] proposed a unified framework that accounted for both denoising and dehazing. They jointly estimated the transmittance and scene radiance by adopting a semantic-guided regularization and transmittance-aware regularization. Specifically, the former was used to ensure the smoothness and edge-preservation in the transmittance, whereas the latter was used to preserve fine details and reduce noise in the scene radiance.

**Figure 9:**
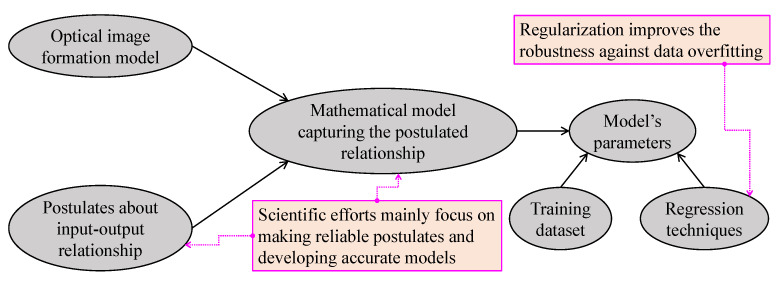
Simplified block diagram summarizing regression and regularization-based visibility restoration methods.

Figure 9 summarizes the most basic steps in regression and regularization-based visibility restoration algorithms. In this approach, researchers typically begin with postulates regarding the input–output relationship, and then develop a corresponding mathematical model. Subsequently, regression techniques can be applied to estimate the model’s parameters. However, this type of parameter estimation is prone to data overfitting; hence, regularization techniques can improve the robustness against this problem. Additionally, improvements in this branch of visibility restoration algorithms mainly lay in making reliable postulates and developing accurate mathematical models.

**Probabilistic graphical model:** unobserved variables of the simplified Koschmieder model exhibit conditional dependence that can be expressed by a probabilistic model encompassing the properties of factorization and independence. This type of modeling technique is beneficial for the analysis of complex data distributions, because it results in a succinct description favoring the extraction and utilization of underlying regularities. Nan et al. [70] improved the simplified Koschmieder model by including the zero-mean Gaussian noise. Subsequently, they devised a Bayesian framework for estimating the transmittance and scene radiance. Despite the high computational cost, the dehazed results were unimpressive when compared to that of contemporary methods. Wang and Fan [71] investigated the effects of the patch size on the estimated depth information and proposed a Bayesian approach for multiscale depth fusion. In this context, prior depth information was calculated at different scales, and they adopted the Markov random field (MRF) to describe the relation between multiscale priors and scene depth. They also adopted the adaptive truncated Laplacian potential to construct the local regularization, which accounted for both smoothing and edge-preserving constraints. However, this method was inefficient, owing to its cubic-time complexity, and the results were affected by color distortion. Similarly, Qu et al. [72] improved the MRF model by considering local pixel blocks with the same depth change, instead of adjacent pixels, as utilized by Wang and Fan [71]. High algorithmic complexity and color distortion persisted, despite the aforementioned improvement.

**Searching-based optimization and linear approximation:** another approach is to leverage searching algorithms to seek the plateau of the induced energy term in visibility restoration. Ju et al. [73] proposed the gamma correction prior (GCP) for stabilizing the scattering coefficient. Subsequently, they exploited GCP to create a virtual transformation image, which was used jointly with the simplified Koschmieder model to derive the formula for scene radiance. The only unknown parameter therein was the scattering coefficient ratio, and they adopted the global-wise Fibonacci search algorithm to estimate it. The results were impressive without noticeable artifacts. Ngo et al. [74] formulated an objective function that conveyed essential image features, such as contrast and sharpness. Therefore, they adopted the Nelder–Mead direct search algorithm to seek the optimum transmittance that maximized the objective function. This method delivered good dehazing performance at the cost of prolonged execution time. Wang et al. [75] proposed a hazy image decolorization method that included the hazy weather effects in the traditional decolorization model. Hence, the induced optimization was non-linear, and the Huber loss was exploited for linear approximation. This method effectively preserved the global luminance while demonstrating good color contrast in grayscales images.

**Other directions:** other machine learning techniques, such as blind source separation, clustering, and dimension reduction, have been applied to visibility restoration. On the one hand, Namer et al. [76] exploited independent component analysis to devise a blind estimation scheme for the polarization degree. Their proposed method overcame the existing problem of contemporary polarimetric dehazing algorithms, which was, the need for sky region presence in the estimation of the polarization degree. Hence, the long-term objective towards the automation of polarimetric dehazing was partly facilitated by the work of Namer et al. [76]. On the other hand, He et al. [77] adopted dictionary learning based on the difference-structure-preservation prior for refining the transmittance, which was predicted while using the least square estimation. Although the dehazing performance was impressive, the execution was highly time-consuming. Chen et al. [78] exploited a set of radial basis functions, whose summation was typically used to approximate a given function and construct a simple neural network for image dehazing. The number of hidden neurons was flexible, depending on the scene complexity. Accordingly, more neurons were utilized for a textual surface and vice versa. Consequently, the run-time was considerably prolonged because the network configuration had to be determined for individual image patches.

Yuan and Huang [79] leveraged image retrieval to obtain external knowledge on the scene being recovered. A feature detection technique that was known as scale-invariant feature transform was adopted at two different scales to retrieve the correlated haze-free references from the database. Global geometric registration and block-based adjustment were then performed to obtain well-registered regions between each image pair, which was, the input image and individual retrieved references. The medium transmittance was estimated using the reference blocks, and Laplacian-based interpolation and regularization were adopted to obtain the whole transmittance. This method provided good results at the cost of high complexity and a lack of generality. Berman et al. [80] proposed a non-local haze-line prior, which stated that a few clusters in the RGB space could approximate the real color of haze-free images. They adopted the *k*-means clustering technique to derive the haze lines and leveraged the *k*-dimensional tree to reduce the run time. Haze lines were used to estimate the transmittance and atmospheric light to recover the scene radiance. This novel prior was exploited in several applications, including underwater color restoration [81], maritime surveillance [82], and three-dimensional (3D)-TV rendering [83].

**Figure 10:**
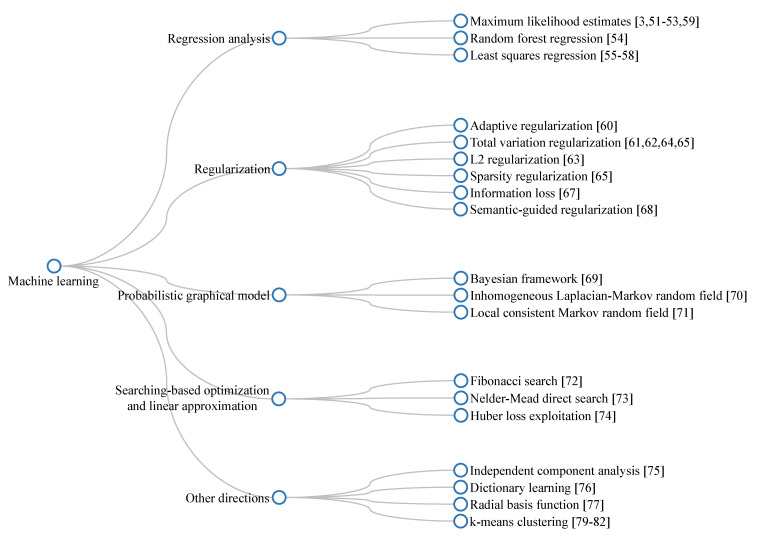
Branching diagram summarizing machine-learning-based dehazing algorithms.

Similar to the previous subsection, a branching diagram shown in Figure 10 provides a quick overview of the aforementioned introduction of dehazing algorithms utilizing machine learning techniques.

#### 2.3.3. Deep Learning

**Convolutional neural network (CNN):** in an artificial neural network, each neuron in a particular layer is typically connected to all neurons in the next layer, hence, the name fully connected network. This type of neuron interconnection may result in overfitting and impede the development of deep neural networks. Accordingly, CNNs can be considered as the regularized versions of fully connected networks because they are useful in avoiding overfitting and reducing interconnections. Inspired by biological processes, CNNs have been developed to resemble the organization of the visual cortex [84]. The response of individual cortical neurons is driven by a restricted region of the visual field, and this region is referred to as the receptive field. Similar to the image filtering technique, the receptive fields of constituent neurons overlap each other to cover the entire visual field. CNNs take advantage of this connectivity pattern; hence, they can extract the hierarchical pattern in the data and combine simpler patterns into more complex patterns. Therefore, they are widely exploited in diverse computer-vision applications, such as image classification and image restoration.

Cai et al. [85] leveraged a three-layer CNN, which is known as DehazeNet, to estimate the medium transmittance. This architecture was quite efficient and straightforward, in which the first layer extracted low-level features from a single input image. The second layer processed these features at different scales to achieve spatial invariance. The last layer combined the previous results into the transmittance. Later, the DehazeNet architecture was exploited in several studies, owing to its simplicity and efficacy. Wang et al. [86] investigated color images in the YCbCr color space and discovered that haze primarily affected the luminance channel. Subsequently, they devised a DehazeNet-like CNN for dehazing only the Y channel; hence, this network was lightweight while retaining comparable performance. Dudhane and Murala [87] furthered the previous work by utilizing two DehazeNet-like CNNs for estimating two versions of the medium transmittance in RGB and YCbCr color spaces. Therefore, they fused the two transmittance estimates using a fusion network to obtain a final transmittance. Recently, Huang et al. [88] devised a dual-subnet network for the joint learning of visibility enhancement, object recognition, and object localization. The restoration network that was employed in that study followed the DehazeNet architecture with three main processes: feature extraction, multiscale mapping, and nonlinear regression. However, the foregoing networks demonstrated average performance, owing to the lack of real training datasets and the simplicity of the employed loss function (that is, mean squared error (MSE)).

Ren et al. [89] utilized a coarse-scale CNN with large filtering windows and a fine-scale CNN with small filtering windows for estimating the transmittance in a multiscale manner. This method differed from the aforementioned methods in the following aspect: the cascaded estimation in coarse-to-fine scale replaced the parallel multiscale mapping. Although the results were generally superior to that of contmpeorary algorithms, this method was affected by the domain shift problem. In this context, two CNNs were trained with a synthetic dataset that was created using the simplified Koschmieder model for homogeneous lighting conditions. Therefore, it failed to restore scene visibility under heterogeneous lighting conditions (for example, nighttime scenes). Additionally, the transmittance estimate was occasionally inaccurate, which resulted in different transmittance values for pixels within the same object. Accordingly, Ren et al. [90] addressed their own limitations by adopting an additional CNN known as the holistic edge guided network to enforce the transmittance smoothness inside the same object. Yeh et al. [91] exploited image decomposition to visibility restoration by dehazing the base layer and enhancing the detail layer. The dehazing task leveraged the multiscale network that was developed by Ren et al. [89] for structural feature extraction and the encoder—decoder framework for statistical feature extraction. These results were fetched to a regression network to obtain the dehazed base layer. For the sharpness enhancement task, a lightweight CNN was utilized to predict the scaling factor. The results demonstrated a satisfactory performance on thin haze scenes, but a lack of qualitative results on moderate and thick haze scenes impeded a complete assessment.

**Figure 11:**
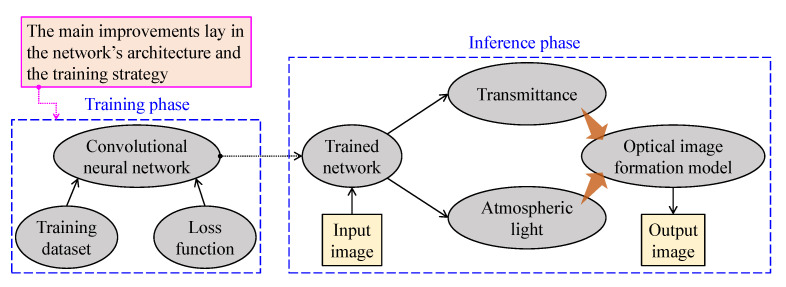
Simplified block diagram of convolutional neural network-based visibility restoration algorithms.

Figure 11 provides a general insight into CNN-based visibility restoration methods. This approach consists of two phases—training and inference—and the main improvements lay in the network’s architecture and the training strategy. At the training phase, researchers develop the network and determine the training strategy—supervised, unsupervised, or hybrid learning method. When the loss function has been successfully settled at a plateau, the trained network is ready for inference. CNN-based methods mainly aim to estimate the medium transmittance and atmospheric light, imposing a performance limit, owing to the simplification of the optical image formation model.

**Generative adversarial network (GAN):** another deep learning framework, known as GAN designed by Goodfellow et al. [92], has been used extensively in visibility restoration. The fundamental idea is based on the competition between a generator and a discriminator in the training phase. The generator (that is, the network to be deployed in the inference phase) learns to generate new data with similar statistics as the given training dataset. Meanwhile, the discriminator is dynamically updated to distinguish the data produced by the generator from the true data distribution. Hence, the generator’s training goal is to minimize the distance to a given dataset and deceive the discriminator into misinterpreting its output as true data. Liu et al. [93] adopted this framework to estimate the medium transmittance from a set of feature maps, including RGB, dark channel, haze-line, and structural features. However, the dehazing performance was unimpressive, because the fully connected generator was relatively straightforward. Accordingly, an efficient encoder–decoder architecture has been exploited. Ren et al. [94] developed a GAN for video dehazing that used a stack of five consecutive frames to predict three central estimates of the transmittance in the frame stack. They adopted an additional semantic segmentation network to enforce smoothness within the same object. This network demonstrated an acceptable performance for video dehazing and it was considered relatively fast when compared to other networks. However, the processing speed of approximately eight frames per second (fps) is inappropriate for real-time processing, which requires at least 25 fps.

Moreover, Li et al. [95] designed a robust dehazing network by exploiting the encoder–decoder-based GAN and semi-supervised learning framework. The designed GAN was trained with two branches sharing the network weights. The first branch followed supervised learning with a labeled synthetic dataset, and the second branch followed unsupervised learning with only real hazy images. The loss function was comprised of supervised losses (MSE, perceptual, and adversarial losses) and unsupervised losses (dark channel and TV losses). Despite the sophisticated network design, the results exhibited ringing artifacts on dense haze scenes. Zhu et al. [96] proposed a compositional-adversarial network, known as DehazeGAN, which embodied multiscale feature extraction and patch-based discrimination. The former was attained via a generator comprising a coarse-scale network and a fine-scale network for extracting multiscale image features that were used to estimate the atmospheric light and transmittance. The latter was attained via a deeply supervised discriminator that was trained to classify individual patches in the input image instead of the entire image. Additionally, this network generated predictions at each convolutional layer to provide the multiple level supervision to train the DehazeGAN. Despite impressive results on synthetic images, DehazeGAN exhibited average performance on real scenes, which was probably caused by the domain shift problem.

Recently, Li et al. [97] contributed significantly to the rapid development of GAN-based visibility restoration. They developed a hybrid network that was based on the encoder–decoder framework and spatially variant recurrent network architecture. This deep neural network was trained using the combination of L1, TV, and dual composition losses to perform the following operations: haze removal, haze residual removal, and image fusion. Despite the expensive computation and supervised learning on a synthetic dataset, this network demonstrated impressive results with high similarity to the ground truth. It was also not affected by the domain shift problem. Apart from the conventional discriminator, Pan et al. [98] introduced a discriminator that was trained to assess the consistency between the regenerated result and the input image. The regeneration process was devised as an inversion of a physical model describing optical image formation. Hence, this framework was highly versatile, because it was applicable to several low-level image restoration tasks. The performance of this physics-based GAN framework largely depended on the physical base model. Accordingly, it is ineffective against images that are degraded by complicated phenomena (for example, heterogeneous light conditions) whose accurate models are currently unavailable. Park et al. [99] developed a heterogeneous GAN that takes advantage of a cycle-consistent GAN (CycleGAN) and a conditional GAN (cGAN) via a fusion CNN. Zhu et al. [100] proposed CycleGAN to enable the training scheme without the strict requirement of a paired dataset through a cycle-consistent loss function. However, CycleGAN had limitations, such as artifacts in dense haze regions and a loss of details. In contrast, Sohn et al. [101] developed cGAN to stabilize the GAN training. Park et al. [99] exploited this framework to estimate the atmospheric light and transmittance, enabling image dehazing through the simplified Koschmieder model. cGAN-based dehazing preserved the fine details in the recovered images, but might suffer from the domain shift problem. Hence, the fusion CNN was used to balance the untoward side effects of CycleGAN and cGAN, producing satisfactory results.

**Figure 12:**
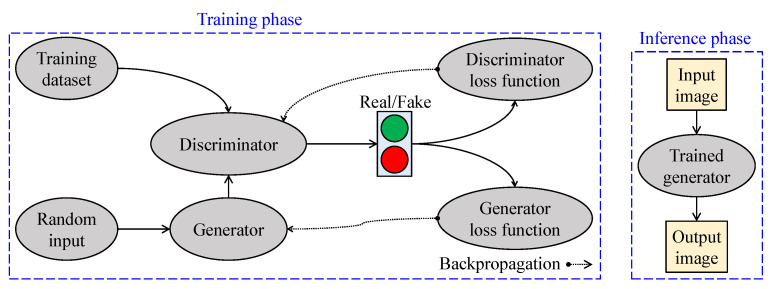
Simplified block diagram of generative adversarial network-based visibility restoration algorithms.

Figure 12 depicts essential aspects of GAN-based visibility restoration algorithms. At the training phase, two networks—generator and discriminator—are utilized to conform with the adversarial training strategy. The generator generates data from a random input, and the discriminator, in turn, attempts to discriminate those data from the real data. The results are then backpropagated to train these two networks. At the inference phase, only the trained generator is deployed in real-world applications.

**Other directions:** Santra et al. [102] proposed estimating the transmittance in local patches based on the quality of the dehazing process. In this context, several dehazed patches were generated using different transmittance values. A CNN that was designed for patch quality comparison was then utilized in combination with a binary search to determine the optimum transmittance value. This method was computationally inexpensive, owing to its simple CNN architecture. However, the results depended considerably on environmental illumination. Golts et al. [103] proposed an unsupervised learning scheme for image dehazing, in which the DCP was exploited to formulate the loss function. Despite improving the traditional DCP and eliminating the need for a paired dataset, this method failed to recover dense haze regions and it exhibited color distortion in the sky regions. Liu et al. [104] attempted to bridge the gap between knowledge-driven and data-driven methods while using a data-and-prior-aggregated transmission network (DPATN). The DPATN fused two transmittance estimates, one from a deep CNN and another from a generalized formula of the DCP, to obtain the final transmittance. The DPATN demonstrated impressive dehazing performance, even in distant regions; however, it might suffer from post-dehazing artifacts. Currently, Li et al. [105] exploited zero-shot learning to devise a training-free unsupervised dehazing network. They utilized three encoder–decoder-based submodules, known as J-Net, T-Net, and A-Net, corresponding to three unknowns in the simplified Koschmieder model. The loss function was minimized using the sole input image, and the dehazed result was the J-Net output. Despite the impressive performance, the inference is time-consuming, because zero-shot learning is still in its infancy and it requires future scientific efforts.

**Figure 13:**
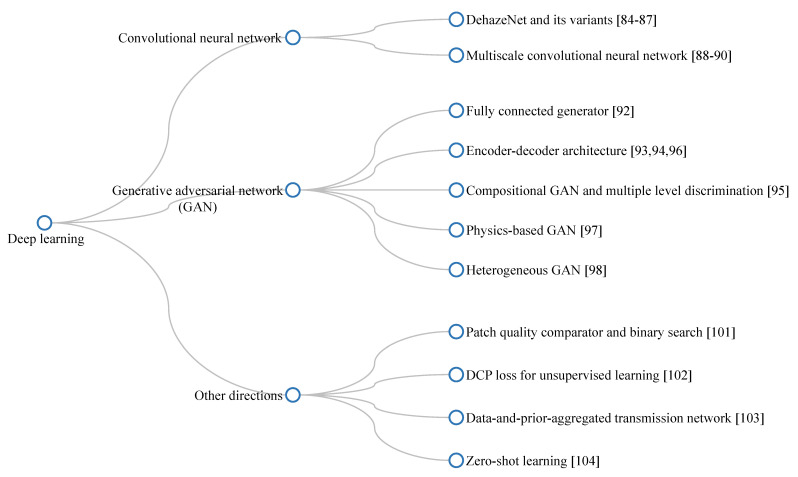
Branching diagram summarizing deep-learning-based dehazing algorithms.

Figure 13 provides a summary of deep-learning-based dehazing algorithms. Additionally, Table 1 provides a quick overview of three main categories of visibility restoration algorithms. As the detailed description is available in the main text, Table 1 presents the most general information on each category and its constituent techniques.

## 3. Current Difficulties

Visibility restoration is an active research area attracting diverse scientific efforts, owing to its ill-posed nature. Although various algorithms covering manifold approaches have been proposed, difficulties that hinder the current progress of visibility restoration persist. Accordingly, this section describes three main issues worthy of collaborative effort.

### 3.1. Real-Time Processing

An image processing algorithm would be a workable solution to a particular problem if it could meet the real-time processing requirements, which is, the capability to handle at least 25 fps and fit into an end-device with limited computing resources. Similar to our previous research [54], six dehazing methods corresponding to three main categories were selected to evaluate the processing time. This experiment was conducted on a computer with an Intel Core i9-9900K (3.6 GHz) CPU, 64 GB RAM, and NVIDIA TITAN RTX GPU. Because all of the methods involved were publicized in the MATLAB source code, MATLAB R2019a was used as the simulation environment. The results shown in Table 2 demonstrate that none of the methods could handle images in real time. The fastest algorithm that was proposed by Kim et al. [36] can process a 640×480 image at 6.25 fps (=1/0.16) and a 4096×2160 image at 0.21 fps (≈1/4.81). This processing speed is far below the real-time requirement of 25 fps, whih suggests that the software implementation is quite impracticable, despite the quick development time.

Moreover, software implementation appears to be inappropriate for real-time visibility restoration because this type of image processing algorithm is typically considered a pre-processing step for high-level computer-vision tasks. Accordingly, it is strictly constrained by the processing time and computing resource utilization. However, the porting of visibility restoration algorithms to target end-devices requires expensive computing elements, owing to the floating-point computations. Even though the source code can be converted to fixed-point representation in advance, the porting is still inefficient. Hence, the hardware implementation is a viable alternative, and FPGA prototypes have garnered increasing interest, owing to their programmability. Shiau et al. [106], Zhang and Zhao [107], and Ngo et al. [54,108] presented typical hardware implementations of visibility restoration algorithms. It is currently observed that image-processing-based and machine-learning-based algorithms favor the hardware implementation phase, whereas deep-learning-based algorithms hinder the realization of their hardware counterparts. In the literature, Eyeriss [109] and its successor [110] provided an energy-efficient framework for designing deep neural networks. However, they primarily favor detection or classification tasks, and attaining real-time processing is still challenging. The facilitation of the hardware implementation of deep-learning-based algorithms is an active research area. Recent efforts include fpgaConvNet, Caffeine, and CNN2Gate that were developed by Venieris and Bouganis [111], Zhang et al. [112], and Ghaffari and Savaria [113], respectively. These frameworks facilitated FPGA prototypes of deep neural networks that were designed using well-known libraries, such as PyTorch and Caffe. Nonetheless, the optimization of hardware resource utilization and real-time processing are challenging problems that require conscious effort.

### 3.2. Training Dataset

**Figure 14:**
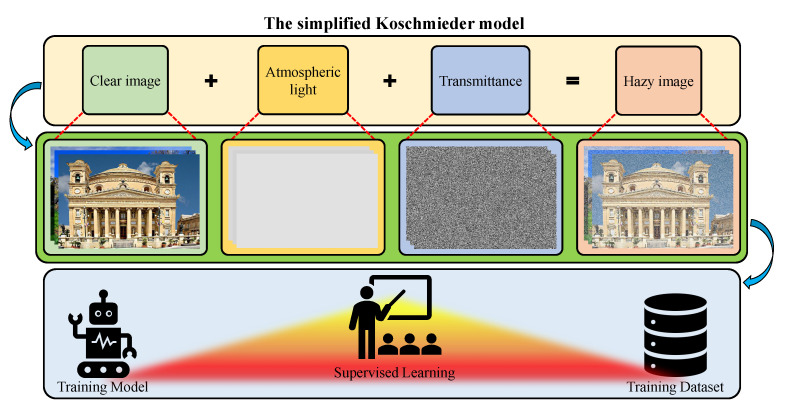
Typical procedure for preparing the synthetic training dataset.

With the increasing research trend towards data-driven algorithms, the role of the training dataset has become crucial. Specifically, in the field of visibility restoration in adverse weather conditions, the acquisition of a reliable dataset appears to be unattainable because capturing the same scene under different weather conditions is impossible. Accordingly, researchers have circumvented this challenging issue by utilizing synthetic training datasets. Figure 14 illustrates a procedure for creating a synthetic training dataset that is based on the simplified Koschmieder model. Clear images are widely available in image-sharing services such as Google Images or Flickr. Hence, researchers have utilized pseudo-random number generators to draw the atmospheric light and medium transmittance (or equivalently the scene depth) from an assumed distribution (for example, uniform or Gaussian). Subsequently, they have substituted those quantities into Equation (5) to obtain the hazy synthetic images. This procedure has been employed in several machine-learning-based and deep-learning-based methods, for example, those that were proposed by Zhu et al. [52], Tang et al. [55], Cai et al. [85], and Ren et al. [89,90].

As the imaging technology advances, depth cameras and stereo cameras have been leveraged to capture the scene depth, partly facilitating synthetic datasets. The NYU Depth v2 dataset that was instituted by Silberman et al. [114] comprises indoor images with their corresponding scene depths captured by the Kinect camera. This dataset has been widely employed in the literature to train machine-learning-based and deep-learning-based models, for example, the task-oriented dehazing network that was designed by Li et al. [97]. Moreover, specialized vapor generators have come into practice to resemble optical hazy image formation. In this manner, Ancuti et al. [115,116,117] instituted three real datasets covering indoor, outdoor, and indoor-outdoor images. Although these datasets appear to be usable in training deep neural networks, the fundamental difference in suspended particle diameters may cause the domain-shift problem. As a result, the preparation of training datasets remains a challenging problem worthy of further research. Before discussing another aspect, this paper tabulates datasets introduced thus far in Table 3 for easy reference.

Other scientific attempts are to alleviate the strict requirement of paired datasets in supervised learning. The semi-supervised learning that is presented by Li et al. [95] trains the network with two different branches, including a supervised branch with a paired dataset and an unsupervised branch with only real data. The performance of the unsupervised learning branch depends on the loss functions. Accordingly, Li et al. [95] leveraged the dark channel’s sparsity and TV to enforce the network to generate images with similar statistical properties as clean images. Golts et al. [103] also exploited the dark channel’s sparsity to devise a wholly unsupervised model for single-image dehazing. However, image artifacts might affect the results, as interpreted by Li et al. [95] in their analysis of the unsupervised loss functions. Ignatov et al. [118] introduced weakly supervised learning, in which the output image was converted back to the input domain via an additional generator for comparison with the input image. The VGG-19 network [119] was utilized to form the content loss function in the input domain. This loss function was then combined with the output loss functions to jointly train the network. The physics-based GAN [98] discussed earlier also followed this weakly supervised learning scheme. Shao et al. [120] adopted the same unsupervised loss function as Li et al. [95] and exploited image translation to bridge the gap between real and synthetic domains. Their work demonstrated promising results in overcoming the domain-shift problem. Despite significant efforts thus far, future research into training dataset preparation and learning schemes is deemed to be a crucial requisite for improving the restoration quality.

### 3.3. Image Formation Model

Image formation in a particular environment is a complex phenomenon that involves various factors, such as the lighting conditions, medium characteristics, object properties, and imaging sensor attributes. The simplified Koschmieder model described earlier was devised based on several assumptions, for example, the atmosphere was homogeneous, and the scattering of reflected light was insignificant. As a result, the majority of visibility restoration algorithms demonstrated poor performance in heterogeneous conditions. Although studies addressing this challenging problem did exist, they were application-specific. Hu et al. [82] tackled glow-shaped environmental illumination in sea fog images while using image decomposition. Similarly, Chiang and Chen [22] employed foreground/background segmentation to deal with artificial light sources in underwater images. Nevertheless, extending these methods to a general case is nontrivial and requires further effort.

Additionally, noise within the digital camera workflow also affects digital image formation. This untoward phenomenon merits consideration. In the literature, Lee et al. [57] considered the demosaicking artifacts and sensor noises to devise a robust algorithm. Wu et al. [69] addressed the noise amplification problem by removing haze and noise in a joint recovery scheme. Despite the efficacy of noise and artifact suppression, these methods failed to consider the heterogeneous transmission medium. Accordingly, an accurate model describing optical image formation is still in great demand. This model will benefit diverse image restoration tasks and alleviate the current problem in training datasets. In this context, an accurate model provides an efficient tool for synthesizing degraded images, which can be used to build a reliable paired dataset for supervised learning.

## 4. Proposed Dehazing Framework

An effective algorithm for visibility restoration in poor weather conditions is still in great demand, as witnessed by the aforementioned review. Knowledge-driven methods can yield satisfactory results, but it may fail in particular circumstances (for example, scenes with a big sky). Similarly, data-driven methods can also produce passable results, but they may be prone to the domain-shift problem. Hence, this section presents a machine-learning-based framework that can balance the untoward effects of knowledge-driven and data-driven methods.

**Figure 15:**
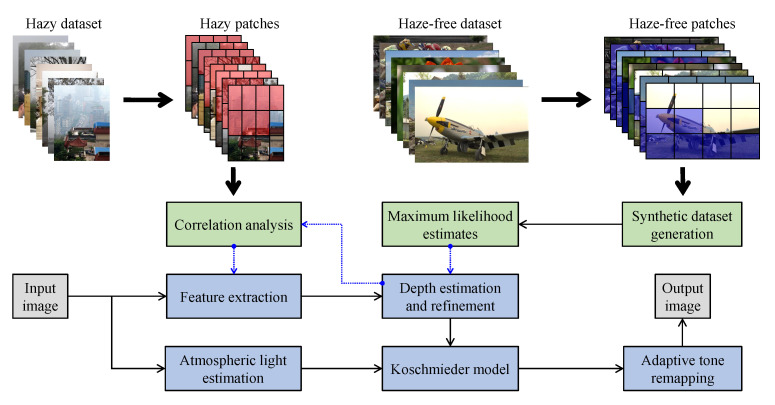
Proposed dehazing framework based on a machine learning technique.

Figure 15 illustrates the diagram of the proposed framework, in which green blocks denote offline computations and blue blocks denote online computations. This framework generalizes the work that was presented by Zhu et al. [52] in color attenuation prior by considering the scene depth estimation with several haze-relevant features. First, to address the domain-shift problem, the hazy and haze-free datasets were processed by a data cleaning step to solely extract the representative hazy and haze-free patches. Subsequently, the haze-free patches underwent the procedure depicted in Figure 14 to create a paired dataset that was used in a supervised learning scheme (that is, MLE) to estimate the scene depth estimator’s parameters. The hazy patches were jointly employed with the depth estimator to determine features that were most pertinent to haze. The online computations were principally similar to those that were utilized by Zhu et al. [52] with the following distinctions. The efficient quadtree-decomposition algorithm [121] and the modified hybrid median filter [36] were utilized for atmospheric light estimation and scene depth refinement. Additionally, adaptive tone remapping [122] was exploited to post-process the dehazed image, restoring the image vividness. The following subsections will describe these issues.

### 4.1. Data Cleaning Based on Haze-Relevant Features

It is observed that hazy images can contain haze-free regions that are generally located near the camera. In contrast, haze-free images can contain regions that are hazy or exhibit characteristics that are similar to haze. For example, clouds or white objects may be misinterpreted as haze, owing to the high similarity in their appearance. As a result, using the entire images or their extracted patches to train the machine-learning or deep-learning models is subject to the domain-shift problem and may result in inaccurate training. This study adopted the data cleaning method that was proposed by Choi et al. [41] to solely extract representative hazy/haze-free patches from the corresponding hazy/haze-free images. In their work, Choi et al. [41] employed 12 haze-relevant features denoted as $f_{i}$, where i∈Z∩[1,12]. The data cleaning step aimed to select image patches that maximized the amount of information conveyed by haze-relevant features. For a particular feature $f_{i}\left(k\right)$, where k∈Z∩[1,K] and *K* denoted the total number of image patches within an image, min-max normalization was conducted, such that $0\leq f_{i}\left(k\right)\leq 1$. Choi et al. [41] selected representative hazy patches satisfying the condition $f_{i}\left(k\right)\leq {\bar{f}}_{i}$, where ${\bar{f}}_{i}$ denoted the average of the feature $f_{i}$ over all *K* patches. In contrast, they selected representative haze-free patches that satisfied the opposite condition $f_{i}\left(k\right)>{\bar{f}}_{i}$.

**Figure 16:**
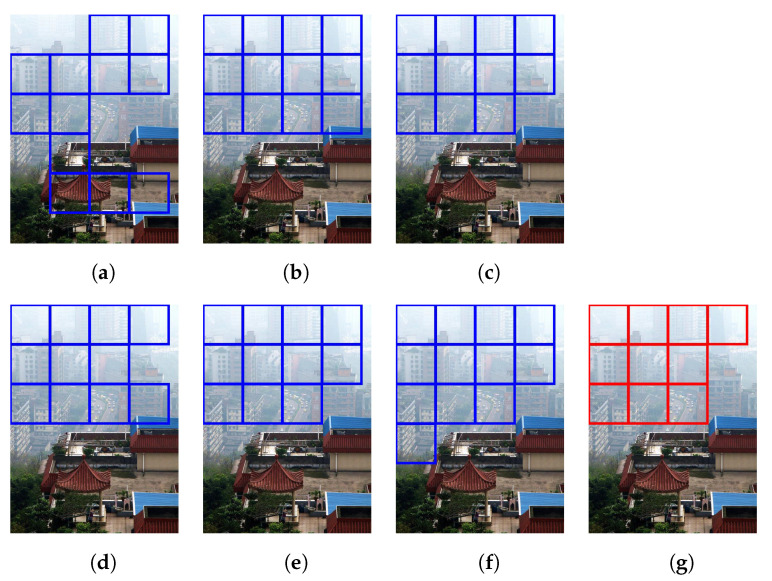
Selection of representative hazy patches based on: (**a**) mean subtracted contrast normalized, (**b**) sharpness, (**c**) contrast, (**d**) entropy, (**e**) dark channel prior, and (**f**) saturation features. (**g**) Selected patches.

**Figure 17:**
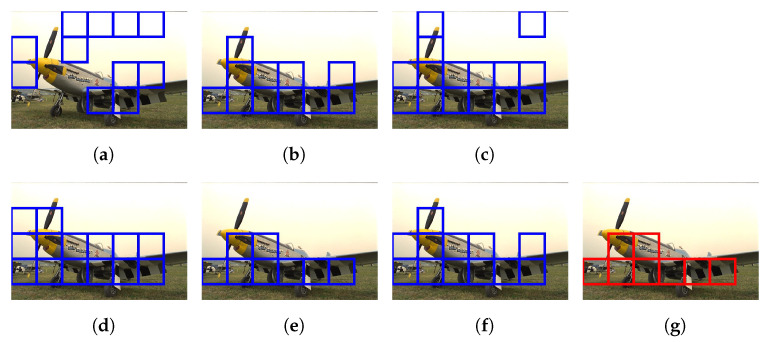
Selection of representative haze-free patches based on: (**a**) mean subtracted contrast normalized, (**b**) sharpness, (**c**) contrast, (**d**) entropy, (**e**) dark channel prior, and (**f**) saturation features. (**g**) Selected patches.

Figure 16 and Figure 17 demonstrate the selection of representative hazy/haze-free patches while using only six haze-relevant features for ease of illustration. The employed features were mean subtracted contrast normalized, sharpness, contrast, entropy, DCP, and saturation. The patch size was set to 111×111. The hazy image that is depicted in Figure 16 contains two distinct regions, which is, a close-field region of a clear rooftop and a far-field region of a hazy city spot. The selected patches that are depicted in Figure 16g were chosen as an intersection of all selected patches using individual features, effectively omitting the haze-free region. Conversely, the haze-free image depicted in Figure 17 contains an immense sky with properties similar to those of haze. Accordingly, the selected patches depicted in Figure 17g only cover the light aircraft and demonstrate the selection scheme’s efficacy.

It is noteworthy that the feature extraction step shown in Figure 15 utilizes the formulas presented by Choi et al. [41] to calculate the haze-relevant features. Therefore, this paper does not rephrase those formulas, and interested readers can refer to Choi et al. [41] for a full description.

### 4.2. Scene Depth Estimation

In the work of color attenuation prior, Zhu et al. [52] postulated that the scene depth closely correlated with the difference between the image saturation and brightness. Therefore, they modeled the scene depth as a linear combination of the image saturation and brightness. This study leveraged the findings of Choi et al. [41] to extend the previous postulate, so that the scene depth could be modeled as a linear combination of all 12 haze-relevant features, as illustrated in Equation (6).

(6) $$d\left(x\right)=\theta_{0}+\sum_{i=1}^{F}\theta_{i}f_{i}\left(x\right)+\varepsilon \left(x\right),$$

where $\theta_{0}$ denotes the bias, $\theta_{i}$ denotes the linear coefficient that is associated with the haze-relevant feature $f_{i}$, *F* denotes the number of features utilized to estimate the scene depth *d*, and $\varepsilon ∼N(0,\sigma^{2})$ denotes the model error following Gaussian distribution with zero mean and $\sigma^{2}$ variance. Accordingly, the scene depth also follows a Gaussian distribution and it can be expressed as $d∼N(\theta_{0}+\sum_{i=1}^{F}\theta_{i}f_{i},\sigma^{2})$. Hence, the linear coefficient $\theta_{i}$ can be estimated by minimizing the model error. For this purpose, Zhu et al. [52] assumed that the random error at individual scene points was independent and identically distributed, consequently resulting in the likelihood *L*, as follows:

(7) $$L=\prod_{j=1}^{N}\frac{1}{\sqrt{2\pi \sigma^{2}}}exp\left\{-\frac{dr_{j}-\left[\theta_{0}+\sum_{i=1}^{F}\theta_{i}f_{i}\left(j\right)\right]}{2\sigma^{2}}\right\},$$

where $dr_{j}$ denotes the ground-truth reference of the scene depth corresponding to the *j*th scene point and *N* denotes the total number of scene points. Minimizing the model error is implicitly attainable by maximizing the likelihood, and it is more convenient to maximize the natural logarithm of the likelihood. The optimization problem is now expressed as

(8) $$\underset{\theta_{0},\theta_{i},\sigma^{2}}{\mathrm{argmax}}ln\left(L\right)=\sum_{j=1}^{N}ln\left(\frac{1}{\sqrt{2\pi \sigma^{2}}}exp\left\{-\frac{dr_{j}-\left[\theta_{0}+\sum_{i=1}^{F}\theta_{i}f_{i}\left(j\right)\right]}{2\sigma^{2}}\right\}\right).$$

Following Zhu et al. [52], the values of $\theta_{0}$, $\theta_{i}$, and $\sigma^{2}$, which maximize the natural logarithm of the likelihood, are given as

(9) $$\begin{array}{ccc} \sigma^{2} & = & \frac{1}{N}\sum_{j=1}^{N}{\left\{dr_{j}-\left[\theta_{0}+\sum_{i=1}^{F}\theta_{i}f_{i}\left(j\right)\right]\right\}}^{2}, \end{array}$$

(10) $$\begin{array}{ccc} \theta_{0} & := & \theta_{0}+\rho \frac{\partial ln(L)}{\partial \theta_{0}}, \end{array}$$

(11) $$\begin{array}{ccc} \theta_{i} & := & \theta_{i}+\rho \frac{\partial ln(L)}{\partial \theta_{i}}, \end{array}$$

where ρ denotes a hyper-parameter known as the learning rate, and the partial derivatives are as follows:

(12) $$\begin{array}{ccc} \frac{\partial ln(L)}{\partial \theta_{0}} & = & \frac{1}{\sigma^{2}}\sum_{j=1}^{N}\left\{dr_{j}-\left[\theta_{0}+\sum_{i=1}^{F}\theta_{i}f_{i}\left(j\right)\right]\right\}, \end{array}$$

(13) $$\begin{array}{ccc} \frac{\partial ln(L)}{\partial \theta_{i}} & = & \frac{1}{\sigma^{2}}\sum_{j=1}^{N}f_{i}\left(j\right)\left\{dr_{j}-\left[\theta_{0}+\sum_{i=1}^{F}\theta_{i}f_{i}\left(j\right)\right]\right\}. \end{array}$$

Notably, the linear coefficients are updated dynamically; hence, the notation := is used in Equations (10) and (11). Moreover, instead of the stochastic gradient ascent algorithm that was employed by Zhu et al. [52], this study exploited the mini-batch gradient ascent algorithm, as described in Algorithm 1. The conditional statement inside the inner loop covers the case when the total scene point *N* is not divisible by the batch size BS. Additionally, the statement “check for termination” determines when to stop the iteration. It jointly tests whether the successive changes in linear coefficients and log-likelihood are below a pre-determined stop criterion. In this study, $10^{8}$ scene points constituted the synthetic training dataset, which was, $N=10^{8}$. The number of epochs EP, batch size BS, learning rate ρ, and stop criterion were set to $10^{5}$, $6\times 10^{5}$, $10^{-8}$, and $10^{-5}$, respectively. Initially, the estimation process involved all 12 features. Subsequently, because each feature exerted a different influence over the scene depth, a correlation analysis was necessary to determine the most pertinent features. The correlation coefficients between individual features and the estimated scene depth were calculated and sorted in descending order using representative hazy patches. According to the number of employed features *F*, the corresponding top *F* features were selected to construct the final model for scene depth estimation. This process reduced the computational burden to a certain extent. In this study, the top four features, including the saturation, brightness, dark channel, and local variance, were selected. The best learning results were obtained after 315 epochs: $\theta_{0}=-0.5770$, $\theta_{1}=0.7243$, $\theta_{2}=-0.3685$, $\theta_{3}=1.5210$, and $\theta_{4}=0.9042$. Finally, the modified hybrid median filter was used to refine the estimated scene depth, enforcing smoothness while retaining edges.
**Algorithm 1** Mini-batch gradient ascent.**Input:** The number of employed features *F*, the training feature vector $f_{i}\in R^{N\times 1}$, the training ground-truth depth vector $dr\in R^{N\times 1}$, the number of epochs EP, the batch size BS, and the learning rate ρ
**Output:** The estimates of $\theta_{0}$, $\theta_{i}$, and $\sigma^{2}$
1: **Initialization** ei=0, bn=⌈N/BS⌉, $\theta_{0}$ and $\theta_{i}$ are initialized with random values drawn from $N(0,10^{-2})$
2: **while** ei<EP **do** 3:  bi=0
4:   **while** bi<bn **do** 5:    **if** bi<bn−1 **then**
6:     $\sigma^{2}=BS^{-1}\sum_{j=bi\times BS}^{(bi+1)\times BS-1}{\left\{dr_{j}-\left[\theta_{0}+\sum_{i=1}^{F}\theta_{i}f_{i}\left(j\right)\right]\right\}}^{2}$
7:     $\theta_{0}=\theta_{0}+\rho \sigma^{-2}\sum_{j=bi\times BS}^{(bi+1)\times BS-1}\left\{dr_{j}-\left[\theta_{0}+\sum_{i=1}^{F}\theta_{i}f_{i}\left(j\right)\right]\right\}$
8:     $\theta_{i}=\theta_{i}+\rho \sigma^{-2}\sum_{j=bi\times BS}^{(bi+1)\times BS-1}f_{i}\left(j\right)\left\{dr_{j}-\left[\theta_{0}+\sum_{i=1}^{F}\theta_{i}f_{i}\left(j\right)\right]\right\}$
9:     check for termination 10:    **else**
11:     $\sigma^{2}={\left(N-bi\times BS\right)}^{-1}\sum_{j=bi\times BS}^{N-1}{\left\{dr_{j}-\left[\theta_{0}+\sum_{i=1}^{F}\theta_{i}f_{i}\left(j\right)\right]\right\}}^{2}$
12:     $\theta_{0}=\theta_{0}+\rho \sigma^{-2}\sum_{j=bi\times BS}^{N-1}\left\{dr_{j}-\left[\theta_{0}+\sum_{i=1}^{F}\theta_{i}f_{i}\left(j\right)\right]\right\}$
13:     $\theta_{i}=\theta_{i}+\rho \sigma^{-2}\sum_{j=bi\times BS}^{N-1}f_{i}\left(j\right)\left\{dr_{j}-\left[\theta_{0}+\sum_{i=1}^{F}\theta_{i}f_{i}\left(j\right)\right]\right\}$
14:     check for termination 15:    **end if**
16:    bi=bi+1
17:   **end while**
18:   ei=ei+1
19: **end while**

### 4.3. Atmospheric Light Estimation

**Figure 18:**
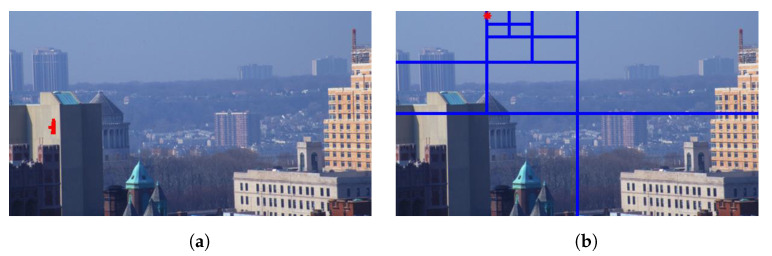
Atmospheric light estimation procedure utilized by: (**a**) Zhu et al. [52]–red pixels belongs to the top 0.1% brightest pixels, and (**b**) Park et al. [121]—blue lines represent the quadtree-decomposition process, and the red dot represents the atmospheric light’s estimate.

According to Equation (5), the atmospheric light is mathematically associated with a pixel at infinite depth, because the transmittance approaches zero as the scene depth goes to infinity. More specifically, d→∞ leads to $t=e^{-\beta d}\to 0$, and Equation (5) yields I=A. However, the practical imaging devices cannot capture scene information at an infinite depth. Accordingly, researchers usually investigate pixels at a considerable distance to estimate the atmospheric light. Those pixels are widely regarded as the most opaque region in the image. He et al. [21] selected the top 0.1% brightest pixels in the dark channel and considered them the most opaque pixels. The atmospheric light was then the pixel with the highest intensity in the input image. Zhu et al. [52] adopted a similar procedure to He et al. [21]. The only difference was that the estimated scene depth was used instead of the dark channel. Despite the widely recognized efficacy, these methods may fail in scenes with bright objects. Figure 18a demonstrated that the method employed by Zhu et al. [52] misinterpreted the bright side of a building as the atmospheric light. The red pixels are the top 0.1% brightest pixels in the estimated scene depth, and they are not the farthest pixels. Accordingly, the estimate of atmospheric light is incorrect in this case, regardless of which pixel among the top 0.1% is selected.

Park et al. [121] presented an efficient algorithm that was based on quadtree-decomposition to address the aforementioned issue. This algorithm divides the input image into quarters and repeats the decomposition in the quarter with the highest average luminance. This process terminates when the quarter size is less than a predetermined value. In the last quarter before termination, the atmospheric light is the pixel with the smallest distance to the white point in the RGB color space. Figure 18b demonstrated that the quadtree-decomposition algorithm that was utilized by Park et al. [121] produced a correct estimate of atmospheric light (that was, the red dot in the upper half). This accuracy is attributed to decomposition that is based on the average luminance. As illustrated in Figure 18b, the bright side of a building is next to the shady side; hence, the average luminance of the corresponding quarter is reduced. Furthermore, the estimated atmospheric light was compensated according to the scheme that was proposed by Ngo et al. [54] to avoid the post-dehazing false enlargement of white objects.

Using the scene depth and atmospheric light estimates, the haze-free image can be recovered through the simplified Koschmieder model. However, this restoration step generally causes overflows and underflows, consequently reducing the image dynamic range. Although a simple tone remapping technique employed by Tarel and Hautiere [35] can solve this problem, only enhancing the luminance channel might cause color distortion. Therefore, this study exploited a more sophisticated method, known as adaptive tone remapping (ATR), which was proposed by Cho et al. [122], to post-process the recovered image. ATR enhances the luminance and then emphasizes the chrominance accordingly to address the color distortion problem. Interested readers can refer to Cho et al. [122] for a clear and concise description.

### 4.4. Evaluation with State-of-the-Art Methods

#### 4.4.1. Employed Datasets

This study employed synthetic and real datasets to assess the proposed dehazing framework and other state-of-the-art benchmark methods. FRIDA2 [123] and D-HAZY [124] are the synthetic datasets. The former includes 66 haze-free images of road scenes, and it is developed for advanced driver-assistance systems using specialized software, known as SiVIC™. Haze-free images are subsequently modified by the simplified Koschmieder model and its variants to create four sets of 66 hazy images, namely homogeneous, heterogeneous, cloudy homogeneous, and cloudy heterogeneous sets. The latter embodies 1472 clear indoor images and their corresponding depth maps that were captured by a Kinect camera. The simplified Koschmieder model was also adopted to synthesize the hazy images.

The real datasets are IVC [125], O-HAZE [115], and I-HAZE [116]. The IVC dataset includes 25 images covering a wide range of objects, such as humans, animals, landscapes, and road scenes. The O-HAZE and I-HAZE datasets include 45 and 30 images of outdoor and indoor spots, respectively. The haze was introduced into clear scenes using a specialized vapor generator. Table 4 presents a summary of the datasets that were employed for algorithm performance evaluation.

#### 4.4.2. Qualitative Evaluation

As the qualitative evaluation is highly subjective, coupled with the fact that all dehazing algorithms can exhibit good results on general outdoor images, visually assessing their dehazing performance is challenging. Accordingly, this subsection provides a comparative assessment using images that may cause untoward results.

**Figure 19:**
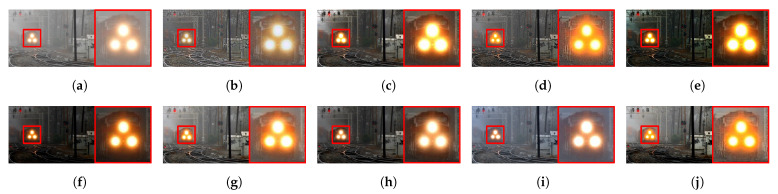
A qualitative comparison of different dehazing methods on a real hazy image of a train. (**a**) Hazy image, and results by (**b**) Tarel and Hautiere [35], (**c**) He et al. [21], (**d**) Kim et al. [36], (**e**) Bui and Kim [50], (**f**) Zhu et al. [52], (**g**) Ngo et al. [74], (**h**) Cai et al. [85], (**i**) Ren et al. [89], and (**j**) the proposed framework.

Figure 19 shows a qualitative comparison of the results with eight typical algorithms that were developed by Tarel and Hautiere [35], He et al. [21], Kim et al. [36], Bui and Kim [50], Zhu et al. [52], Ngo et al. [74], Cai et al. [85], and Ren et al. [89]. The hazy image in Figure 19a is a real scene depicting an approaching train with bright headlights, posing challenges for estimating the atmospheric light. The region of interest was highlighted by a red rectangle, and its enlarged version was exhibited next to the image. A conspicuous problem was the post-dehazing false enlargement of the train headlight, arising in the results by He et al. [21], Bui and Kim [50], and Ngo et al. [74], as depicted in Figure 19c,e,g, respectively. Additionally, the result by Tarel and Hautiere [35] exhibited halo artifacts around fine details and background noises. Kim et al. [36] improved this method to address halo artifacts, but the background noise persisted. Furthermore, the result by Zhu et al. [52] was too dark, and the result of Ren et al. [89] was slightly bluish. DehazeNet, which was designed by Cai et al. [85], produced a satisfactory result, because the haze was removed effectively in both near and distant regions. The proposed framework generated an acceptable result; however, it left more haze in the distant region than DehazeNet.

**Figure 20:**
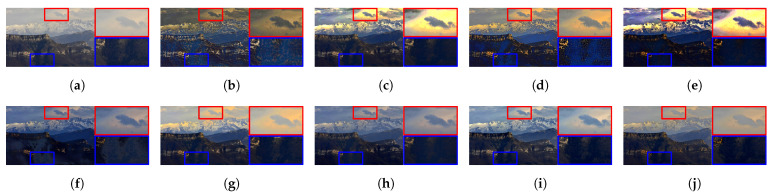
A qualitative comparison of different dehazing methods on a real hazy image of mountains. (**a**) Hazy image, and results by (**b**) Tarel and Hautiere [35], (**c**) He et al. [21], (**d**) Kim et al. [36], (**e**) Bui and Kim [50], (**f**) Zhu et al. [52], (**g**) Ngo et al. [74], (**h**) Cai et al. [85], (**i**) Ren et al. [89], and (**j**) the proposed framework.

Figure 20 illustrates another qualitative comparison. The hazy image shown in Figure 20a is a real hazy scene depicting mountains with bright objects in the background (a snowy mountain and the sky). A practical problem herein is the color distortion, as depicted in the results that were produced by the methods developed by Tarel and Hautiere [35], He et al. [21], Kim et al. [36], Bui and Kim [50], Zhu et al. [52], Cai et al. [85], and Ren et al. [89]. The sky either turned bluish or exhibited untoward colors, and the front mountain turned dark blue. Only the results of Ngo et al. [74] and the proposed framework appeared to be acceptable.

**Figure 21:**
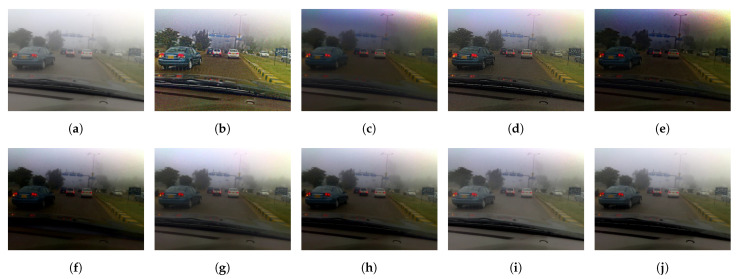
A qualitative comparison of different dehazing methods on a real hazy image of a road scene. (**a**) Hazy image, and results by (**b**) Tarel and Hautiere [35], (**c**) He et al. [21], (**d**) Kim et al. [36], (**e**) Bui and Kim [50], (**f**) Zhu et al. [52], (**g**) Ngo et al. [74], (**h**) Cai et al. [85], (**i**) Ren et al. [89], and (**j**) the proposed framework.

Figure 21 demonstrates the dehazing performance of the proposed framework with that of eight state-of-the-art benchmark methods. The input image shown in Figure 21a is a real road scene covered by a moderate haze. It was observed that the results by Tarel and Hautiere [35], He et al. [21], Kim et al. [36], Bui and Kim [50], and Zhu et al. [52] exhibited color distortion in the sky at different degrees. This problem also existed in the results of Ngo et al. [74], Cai et al. [85], Ren et al. [89], and the proposed framework, but it did not significantly affect the general visibility.

**Figure 22:**
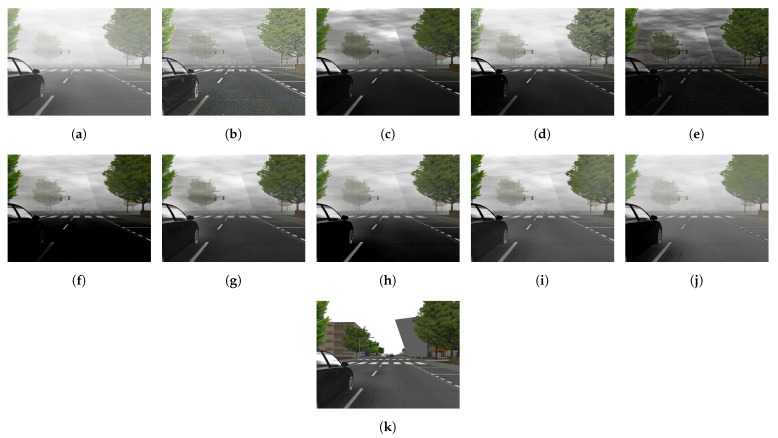
A qualitative comparison of different dehazing methods on a synthetic hazy image of a road scene. (**a**) Hazy image, results by (**b**) Tarel and Hautiere [35], (**c**) He et al. [21], (**d**) Kim et al. [36], (**e**) Bui and Kim [50], (**f**) Zhu et al. [52], (**g**) Ngo et al. [74], (**h**) Cai et al. [85], (**i**) Ren et al. [89], (**j**) and the proposed framework, and (**k**) ground truth.

In addition to the qualitative comparison of real hazy scenes, Figure 22 demonstrates the dehazing performance on a synthetic road scene. Figure 22a,k show the hazy image and the corresponding ground truth. It was observed that the results by Kim et al. [36], Zhu et al. [52], Cai et al. [85], and the proposed framework suffered from the loss of dark details. According to the observation of the car tires and door handles, the method that was developed by Zhu et al. [52] exhibited the highest degree of the loss of dark details, followed by those developed by Cai et al. [85], Kim et al. [36], and the proposed framework, in descending order. Moreover, although the algorithm that was proposed by Tarel and Hautiere [35] demonstrated good visibility, halo artifacts at fine edges posed difficulties for human perception. Additionally, among the impressive results by He et al. [21], Bui and Kim [50], and Ren et al. [89], the result of Bui and Kim [50] exhibited the best visibility. Despite the exceptional dehazing performance, the detailed information of both near and distant objects was well-preserved.

**Figure 23:**
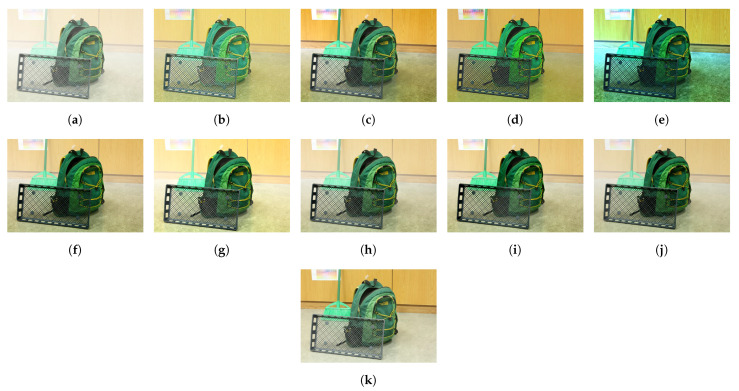
A qualitative comparison of different dehazing methods on a synthetic hazy image of an indoor scene. (**a**) Hazy image, results by (**b**) Tarel and Hautiere [35], (**c**) He et al. [21], (**d**) Kim et al. [36], (**e**) Bui and Kim [50], (**f**) Zhu et al. [52], (**g**) Ngo et al. [74], (**h**) Cai et al. [85], (**i**) Ren et al. [89], (**j**) and the proposed framework, and (**k**) ground truth.

Another qualitative comparison of nine dehazing methods was conducted while using a synthetic image of an indoor scene. Figure 23a,k depict the hazy image and its corresponding ground truth, respectively. Except for the result of Bui and Kim [50], all of the results by other authors and the proposed framework exhibited a satisfactory restoration quality. The result by He et al. [21] demonstrated a high similarity with the ground truth, followed by those of Kim et al. [36], Zhu et al. [52], Ngo et al. [74], and Tarel and Hautiere [35]. In this case, the result by the proposed framework was on par with those by Cai et al. [85] and Ren et al. [94].

A qualitative comparison of different dehazing methods using the real and synthetic images revealed that image-processing-based or machine-learning-based methods tended to produce results favoring human perception. In contrast, deep-learning-based methods exhibited an average performance. This finding might be interpreted, as follows. Image-processing-based and machine-learning-based algorithms were developed from handcrafted features of hazy and haze-free images, resulting from researchers’ manual analyses. Hence, these features were highly perceptible to human visual systems because they typically comprised essential aspects of images, such as contrast, sharpness, and colorfulness. However, deep-learning-based algorithms learnt image features from the training dataset; hence, they were prone to the domain-shift problem. Although image-processing-based and machine-learning-based methods usually favor human visual systems, they may also be beset with noticeable artifacts in unpropitious circumstances.

#### 4.4.3. Quantitative Evaluation

The human subjective assessment is the most accurate method for evaluating the performance of algorithms; however, it is laborious and unrepeatable. Hence, IQA metrics were developed. For the dataset without the corresponding ground truth, such as IVC, this study employed the blind IQA metrics that were proposed by Hautiere et al. [126], known as the rate of new visible edges (*e*) and the quality of contrast restoration (*r*). These two metrics were calculated according to the invisible edges in the original image, which became visible in the restored image. Therefore, higher *e* and *r* values signified better restoration quality. However, notably, Hautiere et al. [126] defined a local-contrast threshold of 5% to determine whether edges were visible. Accordingly, the *e* and *r* metrics were slightly prone to noise, for example, background noises and halo artifacts. Consequently, the quantitative results that are associated with these blind IQA metrics do not necessarily correspond to the qualitative results presented earlier.

For datasets containing ground-truth references, the feature similarity index extended to color images (FSIMc) and the tone-mapped image quality index (TMQI) were adopted to assess the dehazing performance. Zhang et al. [127] proposed the FSIMc to improve the well-known structural similarity index. The TMQI was developed by Yeganed and Wang [128] to assess the dynamic range of the restored image when compared to the ground-truth HDR image. Therefore, high FSIMc and TMQI scores are favorable in the visibility restoration field. Although FSIMc and TMQI are more statistically robust than *e* and *r*, they do not necessarily correspond to the human visual system, because they indeed assess the degradation level.

Table 5 demonstrates the dehazing performance of the proposed framework and the eight benchmark methods on the FRIDA2 dataset. The top three results are boldfaced with red, green, and blue in descending order. The methods of Bui and Kim [50] and Ren et al. [89] exhibited the best performance in terms of TMQI and FSIMc, respectively. Additionally, after poring over the results, this study concluded that deep-learning-based methods demonstrated high quantitative results. Furthermore, there was room for improvement in this case, because the highest score was approximately 0.8, whereas it could reach the ideal value of 1. For the synthetic dataset of the road scene, the proposed framework exhibited comparative performance in terms of FSIMc and slightly poor performance in terms of TMQI.

Table 6 summarizes the average scores of IQA metrics on the IVC, D-HAZY, O-HAZE, and I-HAZE datasets. The top three results are boldfaced in red, green, and blue, in descending order. For the IVC dataset, the method of Bui and Kim [50] exhibited the highest *e* and *r* scores, followed by the methods of Tarel and Hautiere [35] and Kim et al. [36]. Nonetheless, it was observed in the qualitative comparison that these methods were prone to noise and artifacts. These untoward components were misinterpreted as visible edges and subsequently contributed to the high scores of *e* and *r*. Meanwhile, the remaining methods demonstrated a comparative performance. For the synthetic indoor dataset, like D-HAZY, the methods of Bui and Kim [50] and He et al. [21] exhibited the best performance in terms of TMQI and FSIMc, respectively. Because these two methods produced artifacts in the sky, the high performance with the synthetic indoor dataset was explicable. Notably, these findings are consistent with the results that were reported by Ancuti et al. [124]. For the real outdoor and indoor datasets (O-HAZE and I-HAZE), the proposed framework demonstrated the best performance, being on par with the deep-learning-based method of Ren et al. [89]. Despite the impressive results of quantitative comparison, it could only be concluded that the proposed framework possessed a comparative performance, and there was room for future development.

## 5. Conclusions

This study collated information from existing research on visibility restoration in poor weather conditions to identify the current research gaps. The main contribution of this study is the comparison and classification of systematically selected studies. Additionally, the results were tabulated and visualized to effectively transfer knowledge among image processing researchers, practitioners, and laypeople. This study also identified the current difficulties hindering future research, including a lack of real-time processing capability, reliable training datasets, and accurate image formation models.

Section 4 presented a dehazing framework generalizing the color attenuation prior by considering several haze-relevant features. This framework was efficient and it produced comparative results, as demonstrated by a meta-analysis. It was also observed that image-processing-based and machine-learning-based methods produced results favored by human perception. Conversely, deep-learning-based methods were trained by minimizing the measurable distance of statistical regularities between the observed data and ground-truth references. Accordingly, they favored quantitative assessment, as witnessed by high scores of IQA metrics. However, because the IQA metric does not fully represent the human visual system, the results that are produced by early deep-learning-based methods may be less favored by human perception. With the significant advances in learning strategies, the results that are produced by current deep-learning models are of exceptional quality.

The field is slightly stabilizing after reaching its formative stage. This study identified that the expensive computation impeded the broad deployment of deep-learning-based approaches, despite their outstanding performance. It was also observed that automation tools were developed to facilitate the real-time processing of deep-learning models. Nonetheless, the attainable speed was still far below the real-time processing requirement, and resource utilization was not optimized. In conclusion, it is deemed that collaborative efforts are required to develop an accurate image formation model for further enhancement of the field.

## Figures and Tables

**Table 1:** Summary of visibility restoration algorithms in the literature.

Category	Typical Techniques	Pros and Cons
Image processing	Histogram equalization	Pros: Simplicity and fast processing speed
		Cons: Noise amplification
	Polarimetric dehazing	Pros: High restoration quality
		Cons: Complex configuration of experimental equipment
	Dark channel prior	Pros: High restoration quality and efficacy
		Cons: Failures in sky regions
	Image fusion	Pros: Circumvention of challenging estimation process, efficacy, and fast processing speed
		Cons: Tradeoff between restoration quality and hardware friendliness
	Color ellipsoid prior	Pros: High restoration quality and robustness to noise
		Cons: Probable artifacts in dense-haze regions
	Patch similarity	Pros: High restoration quality and versatility
		Cons: Probable ringing artifacts
Machine learning	Regression	Pros: Simplicity and efficacy
		Cons: Data overfitting and poor performance in dense-haze regions
	Regularization	Pros: Robustness to overfitting and high restoration quality
		Cons: Prolonged processing time and probable color distortion
	Probabilistic graphical model	Pros: Facilitation of the analysis of complex data distributions
		Cons: High algorithmic complexity and probable color distortion
	Searching-based optimization	Pros: High restoration quality
		Cons: Prolonged processing time
	Radial basis function	Pros: High restoration quality
		Cons: Prolonged processing time
	Non-local haze-line prior	Pros: High restoration quality
		Cons: Tradeoff between restoration quality and processing time
Deep learning	Convolutional neural network	Pros: Spatial invariance and high restoration quality
		Cons: Poor performance in heterogeneous lighting conditions and probable domain-shift problem
	Generative adversarial network	Pros: High restoration quality
		Cons: Unstable training phase and probable domain-shift problem
	Zero-shot learning	Pros: High restoration quality and elimination of training phase
		Cons: Prolonged inference time

**Table 2:** Processing time in seconds of different dehazing methods for various image sizes.

Category	Method	Image Size				
		640 × 480	800 × 600	1024 × 768	1920 × 1080	4096 × 2160
Image processing	Kim et al. [36]	0.16	0.29	0.43	1.01	4.81
	Bui and Kim [50]	0.32	0.52	0.86	2.37	10.06
Machine learning	Zhu et al. [52]	0.22	0.34	0.55	1.51	6.39
	Ngo et al. [54]	0.18	0.34	0.49	1.13	5.77
Deep learning	Cai et al. [85]	1.53	2.39	3.88	10.68	47.35
	Ren et al. [89]	0.54	0.88	1.53	3.43	17.90

**Table 3:** Summary of usable training datasets mentioned in this section.

Dataset	Authors	Description	Available at
NYUDepth v2	Silbermanet al. [114]	Indoor images and corresponding scene depths captured by Kinect camera	https://cs.nyu.edu/~silberman/datasets/nyu_depth_v2.html (accessed on 19 January 2021)
O-HAZE	Ancutiet al. [115]	Pairs of outdoor real hazy and haze-free images	https://data.vision.ee.ethz.ch/cvl/ntire18//o-haze/ (accessed on 21 January 2021)
I-HAZE	Ancutiet al. [116]	Pairs of indoor real hazy and haze-free images	https://data.vision.ee.ethz.ch/cvl/ntire18//i-haze/ (accessed on 21 January 2021)
Dense-Haze	Ancutiet al. [117]	Pairs of both outdoor and indoor real hazy and haze-free images	https://data.vision.ee.ethz.ch/cvl/ntire19//dense-haze/ (accessed on 21 January 2021)

**Table 4:** Summary of the datasets employed for evaluation. NA stands for not available.

Type	Dataset	Hazy Images (#)	Haze-Free Images (#)	Ground Truth
Synthetic	FRIDA2	264	66	Yes
	D-HAZY	1472	1472	Yes
Real	IVC	25	NA	No
	O-HAZE	45	45	Yes
	I-HAZE	30	30	Yes

**Table 5:** Quantitative evaluation results of different dehazing methods on the FRIDA2 dataset.

Method	Metric	Haze Type ${}^{1}$				
		Type 1	Type 2	Type 3	Type 4	Overall Average
Tarel and Hautiere [35]	TMQI	0.7259	0.7310	0.7312	0.7373	0.7314
	FSIMc	0.7833	0.7725	0.7567	0.8104	0.7807
He et al. [21]	TMQI	0.7639	0.6894	0.6849	0.7781	0.7291
	FSIMc	0.8168	0.7251	0.7222	0.8343	0.7746
Kim et al. [36]	TMQI	0.7320	0.7037	0.7015	0.7343	0.7179
	FSIMc	0.8048	0.7805	0.7751	0.8134	0.7935
Bui and Kim [50]	TMQI	0.7973	0.6956	0.6785	0.8163	**0.7469** ${}^{2}$
	FSIMc	0.8106	0.7057	0.6955	0.8427	0.7636
Zhu et al. [52]	TMQI	0.7533	0.7254	0.7080	0.7674	**0.7385** ${}^{2}$
	FSIMc	0.7947	0.7845	0.7764	0.8117	0.7918
Ngo et al. [74]	TMQI	0.7005	0.6976	0.6867	0.7135	0.6996
	FSIMc	0.7950	0.8014	0.7931	0.8078	**0.7993** ${}^{2}$
Cai et al. [85]	TMQI	0.7398	0.7307	0.7119	0.7592	**0.7354** ${}^{2}$
	FSIMc	0.7987	0.7886	0.7778	0.8199	**0.7963** ${}^{2}$
Ren et al. [89]	TMQI	0.7165	0.7275	0.7094	0.7393	0.7232
	FSIMc	0.8044	0.7922	0.7831	0.8239	**0.8009** ${}^{2}$
Proposed framework	TMQI	0.7027	0.6917	0.6797	0.6707	0.6862
	FSIMc	0.8013	0.7852	0.7890	0.7771	0.7882

${}^{1}$ Types 1, 2, 3, and 4 are homogeneous, heterogeneous, cloudy homogeneous, and cloudy heterogeneous, respectively. ${}^{2}$ The top three results are boldfaced with red, green, and blue in descending order.

**Table 6:** Quantitative evaluation results of different dehazing methods on the IVC, D-HAZY, O-HAZE, and I-HAZE datasets.

Dataset	IVC		D-HAZY		O-HAZE		I-HAZE	
Metric	e	r	TMQI	FSIMc	TMQI	FSIMc	TMQI	FSIMc
Method								
Tarel and Hautiere [35]	**1.30** ${}^{3}$	**2.15** ${}^{3}$	0.8000	0.8703	0.8416	0.7733	**0.7740** ${}^{3}$	0.8055
He et al. [21]	0.39	1.57	**0.8631** ${}^{3}$	**0.9002** ${}^{3}$	0.8403	**0.8423** ${}^{3}$	0.7319	0.8208
Kim et al. [36]	**1.27** ${}^{3}$	**2.07** ${}^{3}$	**0.8702** ${}^{3}$	0.8590	0.6502	0.6869	0.7026	0.7879
Bui and Kim [50]	**1.80** ${}^{3}$	**2.37** ${}^{3}$	**0.8799** ${}^{3}$	0.8554	0.7655	0.7576	0.7116	0.7737
Zhu et al. [52]	0.78	1.17	0.8206	**0.8880** ${}^{3}$	0.8118	0.7738	0.7512	0.8252
Ngo et al. [74]	0.53	1.29	0.7683	0.8676	**0.8616** ${}^{3}$	0.8244	**0.7756** ${}^{3}$	**0.8522** ${}^{3}$
Cai et al. [85]	0.63	1.28	0.7932	**0.8870** ${}^{3}$	0.8413	0.7865	0.7601	0.8482
Ren et al. [89]	0.65	1.47	0.8021	0.8821	**0.8645** ${}^{3}$	**0.8402** ${}^{3}$	0.7719	**0.8521** ${}^{3}$
Proposed framework	0.62	1.55	0.7668	0.8565	**0.8938** ${}^{3}$	**0.8277** ${}^{3}$	**0.8006** ${}^{3}$	**0.8618** ${}^{3}$

${}^{3}$ The top three results are boldfaced with red, green, and blue in descending order.

## Data Availability

Data available in a publicly accessible repository The datasets presented in this study are openly available in [41,114,115,116,117,123,124,125].

## Abbreviations

The following abbreviations are used in this manuscript:

PRISMA	Preferred Reporting Items for Systematic Reviews and Meta-Analyses
A/D	Analog-to-Digital
RGB	Red-Green-Blue
FPGA	Field-Programmable Gate Array
DCP	Dark Channel Prior
GIF	Guided Image Filter
WGIF	Weighted Guided Image Filter
G-GIF	Globally Guided Image Filter
NIR	Near-Infrared
HDR	High Dynamic Range
GLIP	Generalized Logarithmic Image Processing
CEP	Color Ellipsoid Prior
MLE	Maximum Likelihood Estimates
IQA	Image Quality Assessment
TV	Total Variation
MRF	Markov Random Field
CNN	Convolutional Neural Network
MSE	Mean Squared Error
GAN	Generative Adversarial Network
fps	Frames Per Second
CycleGAN	Cycle Consistent Generative Adversarial Network
cGAN	Conditional Generative Adversarial Network
DPATN	Data-and-Prior-Aggregated Transmission Network
ATR	Adaptive Tone Remapping
